# Beyond deficit and coexistence: Modeling the knowledge–conspiracy–mistrust configuration in public understanding of science

**DOI:** 10.1371/journal.pone.0341946

**Published:** 2026-09-18

**Authors:** Ahmet Süerdem, Svetlomir Zdravkov, Martin J. Ivanov

**Affiliations:** 1 Istanbul Bilgi University, Istanbul, Türkiye; 2 Institute of Philosophy and Sociology, Bulgarian Academy of Sciences, Sofia, Bulgaria; National Centre for Scientific Research (CNRS) / Universite Clermont Auvergne / Clermont Auvergne INP, FRANCE

## Abstract

Debates about public trust in science often contrast deficit-based models, which emphasize the role of scientific knowledge, with constructivist perspectives that highlight the coexistence of multiple epistemologies. However, both approaches tend to overlook the mechanisms that link scientific knowledge, alternative epistemic orientations, and mistrust in science. To address this gap, the study applies a multilevel structural equation model within a multidimensional framework to examine conspiratorial reasoning as a key mechanism through which scientific knowledge influences science mistrust. Using cross-national survey data from Europe during the COVID-19 pandemic, the analysis also considers how this pathway is moderated by individual cognitive, motivational, and ideological traits, as well as macro-level political, cultural and economic factors. The findings reveal that conspiratorial reasoning significantly mediates the relationship between scientific knowledge and mistrust at both individual and regional levels. Moreover, the strength of these associations is conditioned by factors like informational engagement, regional value climates, and religiosity. Overall, the results suggest that scientific knowledge serves as a conditional epistemic resource, rather than a consistent buffer against mistrust in science.

## I. Introduction

Over decades of research, the concept of Civic Scientific Literacy (CSL) has been developed to capture the minimum level of scientific understanding required for citizens to engage meaningfully in democratic debates about science and technology [1–3]. Basically, CSL comprises four cognitive-attitudinal components - content knowledge, procedural understanding, appreciation of the positive impact of science, and the rejection of superstition. The framework conceptualized these components as tightly aligned and measurable along a single unidimensional scale. This approach was subsequently adopted by major international survey programs, establishing a standardized metric for assessing public understanding of science across nations.

Such unidimensional conception reflects the logic of the Deficit Model (DM), which attributes public mistrust of science to ignorance and assumes that increasing knowledge automatically produces acceptance of scientific institutions. However, this linear view has been widely criticized for ignoring the social and cultural contexts shaping public understanding of science. Wynne [4] showed that lay mistrust often arises not from ignorance but from experiences of institutional unresponsiveness and marginalization, while Jasanoff [5] critiqued technocratic models that privilege elite knowledge and dismiss lay reasoning. These critiques led to Public Engagement with Science (PES) approaches, which stress that perceived ignorance often reflects contextually grounded understanding shaped by identity, values, and lived experience [6].

While PES offered an important corrective to the DM’s reductionism and promoted more participatory science–society relations, it has also generated methodological and analytical limitations. As Irwin [7] observed, the field has fragmented into small-scale qualitative studies that, despite rich local insight, struggle to produce systematic or generalizable patterns. This parochial focus poses two main risks: overlooking structural and cross-cultural factors that shape public engagement and blurring the boundary between expert and lay knowledge [8]. Such conflation complicates the evaluation of validity claims [9] and risks epistemic relativism, where all forms of knowledge are treated as equally authoritative.

Consequently, both the DM and PES share a critical blind spot: neither satisfactorily theorizes how individual components of CSL interact with contextual conditions to shape public understanding of science. PES rightly foregrounds local identities and lived experience, but often conflates individual agency with structural context, making it difficult to disentangle distinct psychological and social mechanisms. In contrast, the DM offers clearer constructs and causal pathways but does so at the cost of analytical reductionism. These limitations highlight the need for an integrative framework that treats CSL as multidimensional and examines how its components interact with both individual cognitive and motivational factors and broader macro-structural contexts.

Recent scholarship has increasingly adopted integrative perspectives on the relationship between scientific knowledge and attitudes, a direction to which this study contributes. While prior research has focused on direct associations between the two, systematic investigation of the indirect mechanisms remains limited. To address this gap, the present study investigates both individual- and macro-level pathways connecting key dimensions of CSL - namely, knowledge, attitudes, and superstition, operationalized here as conspiracy reasoning.

In what follows, we develop a theoretical framework that reconceptualizes CSL as a multidimensional, context-dependent configuration, emphasizing the mediating role of conspiratorial reasoning in linking scientific knowledge to institutional mistrust. We then translate this framework to a multilevel analytical model and derive testable hypotheses capturing both individual- and regional-level pathways. Drawing on cross-national survey data collected across Europe during the COVID-19 pandemic—a period marked by acute epistemic uncertainty and contestation of institutional authority—we examine these relationships using multilevel structural equation modelling. The paper concludes by considering the implications of the findings for broader debates on public understanding of science, epistemic authority, and science–society relations, moving beyond deficit-based and relativist accounts.

## II. Previous research and theoretical consideration

### A. Beyond simple deficits: Revisiting civic scientific literacy components

Recent scholarship increasingly transcends the rigid DM–PES divide. Rather than rejecting deficit-based explanations, scholars now seek to integrate core CSL constructs with context-sensitive models of public engagement. Einsiedel [10], for instance, emphasizes the existence of “multiple publics” and the need to situate CSL within broader social contexts. Methodological and empirical work further shows that quantitative approaches need not be tied to linear deficit assumptions and that attitudes reflect interactions among knowledge, socio-cultural orientations, and institutional contexts [11,12]. Collectively, these contributions have steered the field toward “theories of the middle range” [13], which capture the multidimensional and multilevel character of science–society relations while retaining key explanatory variables.

Within this synthesized framework, knowledge still remains an important predictor of attitudes, retaining independent effects even as contextual factors condition its influence [11,14]. Large-scale multilevel research supports a modest but consistent association between knowledge and favourable attitudes across diverse national contexts [15]. However, this general relationship is not uniform: in contentious contexts, the link often weakens or even reverses. Knowledge’s protective effect appears contingent on the issue at hand, proving less effective in areas characterized by moral controversy or heightened perceived risk [16–18]. For instance, while knowledge may predict positive attitudes toward vaccines and GMOs, it is less consistently related to climate change scepticism or institutional trust [19]. When scientific claims threaten core values or group identities, motivated reasoning can lead to increased polarization, with higher knowledge sometimes aligning with stronger resistance through identity-protective cognition [20].

Furthermore, CSL encompasses not only knowledge and attitudes but also the rejection of superstition, broadly understood to include conspiratorial, pseudoscientific, and denialist epistemologies. Within the literature, these epistemic orientations are frequently described as “unwarranted epistemologies” or forms of “contaminated mindware” [21,22], denoting cognitive and psychological deficits that distort evidence evaluation rather than coherent alternative ways of reasoning. They are typically framed as the antithesis of rational thought, associated with a dogmatic cognitive style characterized by lower openness to experience, resistance to disconfirming evidence, and limited reflective reasoning [23]. Moreover, endorsement of one such belief system often predicts endorsement of others - a pattern labelled monological reasoning, in which interrelated beliefs dogmatically reinforce one another, sustaining a unified but distorted worldview [24,25].

Among these belief systems, the link between conspiratorial thinking and rational reasoning has received substantial scholarly attention in recent years. Lewandowsky et al. [26] found that while motivational factors such as political ideology predict anti-scientific attitudes in domain-specific ways, conspiracy belief consistently emerges as a strong, cross-domain predictor. It has also been associated with deficits in information processing, including increased susceptibility to misinformation [27], as well as with psychological traits such as a heightened need for control and certainty, and, in some cases, paranoia or delusion-like ideation [28,29]. Accordingly, stronger endorsement of conspiratorial beliefs is frequently linked to lower educational attainment, reduced cognitive complexity, weaker analytic reasoning, and broader tendencies toward irrationality [23,29,30].

Although labelling a claim as a *“conspiracy theory”* imposes the stigma of *“crippled epistemology”* [31], effectively marking it as distorted or irrational, empirical evidence shows that conspiratorial beliefs are widespread and not confined to pathological or fringe groups [32]. Meta-analytic reviews reveal only weak or inconsistent associations with stable personality traits once methodological differences are controlled [33,34]. In light of these findings, scholars increasingly conceptualize conspiracy beliefs as counter-authoritative epistemologies [35], interpreting them as culturally and epistemically embedded responses to uncertainty rather than as unequivocal indicators of cognitive or psychological dysfunction [36–38].

Research increasingly highlights the contingent relationship between scientific and alternative epistemologies, especially in non-western contexts. In Japan [39] and Taiwan [40], for instance, higher levels of factual knowledge have been shown to positively predict engagement with pseudoscientific practices - patterns attributed to intellectual curiosity and dialectical cognitive orientations that favour reconciling apparent contradictions. In the Nigerian context, Falade and Bauer [41] interpreted the interaction between religiosity and knowledge in predicting attitudes towards science as suggesting that scientific and religious epistemologies can coexist, with scientific understanding not necessarily contradicting religiosity across its varying intensities.

### B. Epistemic pluralism without relativism: On coexistence, conflict, and the conditions of epsitemic justification

The coexistence of scientific and lay epistemologies is often explained through cognitive polyphasia (CP), which holds that individuals draw on multiple, culturally embedded epistemic frameworks to interpret and legitimize truth claims [42]. From this perspective, commonsense knowledge does not merely oppose science but provides shared meanings that shape identity and help manage uncertainty. Though seemingly irrational to outsiders, such reasoning remains psychologically coherent and socially meaningful to those who employ it [43]. These lay forms of knowing can also resist or reframe scientific meanings, influencing both research agendas and the translation of science into practice [44]. CP thus portrays knowledge production as a dialogical process in which scientific and non-scientific epistemologies coexist and mutually influence one another.

Despite its heuristic appeal, CP faces significant conceptual and methodological limitations if not carefully operationalized. Conceptually, its ambiguous formulation risks becoming a catch-all for epistemic diversity rather than serving as a precise analytical framework for understanding how competing truth claims coexist—or come into conflict. Methodologically, CP is challenged by its discursive and context-sensitive nature, often requiring in-depth qualitative fieldwork and resisting straightforward quantitative operationalization, especially across cultural contexts [45]. This necessitates mixed-methods approaches; however, without rigorous integration, such combinations risk degenerating into ad hoc eclecticism characterized by superficial ethnographic insights and unprincipled statistical interpretations, ultimately undermining both validity and accountability [46]. Overreliance on simplistic quantitative analysis to demonstrate the coexistence of science and religion (or superstition) can obscure the nuanced, contingent dynamics of epistemic interaction, conflating abstract constructs with the situated practices through which individuals negotiate meaning.

Moreover, CP must contend with the uneven power dynamics that shape how truth claims are legitimized and contested. Knowledge is embedded in power relations that influence the reconstruction, recognition and distribution of epistemic authority [46,47]. Any application of CP must therefore go beyond acknowledging plural perspectives to critically interrogate the conditions under which epistemic legitimacy is constructed and maintained. Blurring the boundary between lay and scientific epistemologies can be problematic, as both are internally fragmented and composed of multiple, sometimes conflicting, configurations. Apparent harmonies may reflect only transient alignments produced by contextual asymmetries.

In such fluid epistemic landscapes, epistemic justification becomes precarious, with standards of evidence and rationality varying across clusters—rendering a claim credible in one context but illegitimate in another. Without a reflexive analysis of how justification functions across these boundaries, CP risks conflating coexistence with equivalence, treating incommensurable epistemologies as if they were symmetrical. This leads to a form of implicit relativism that confuses epistemology—how knowledge is justified—with ontology—what exists independently of that knowledge [48,49]. Absent clear criteria for distinguishing warranted knowledge from misinformation or strategic claims, the very standards of epistemic justification risk being eroded.

Indeed, the embodied and intuitive heuristics underpinning commonsense knowledge can expose the limits of scientism and prompt critical reflection on how epistemic authority of the scientific establishment is constructed and mobilized to serve political or private interests. Yet, reliance solely on intuitively salient cues can also distort judgment, with serious consequences for science-informed decision-making - particularly in sensitive domains such as public health [27,50]. In such contexts, appeals to “multiple truths” may function less as genuine expressions of epistemic pluralism than as strategic instruments to obscure evidence, manufacture doubt, or delay policy action. Rather than fostering dialogical engagement between epistemologies, these dynamics often manifest as struggles over credibility and legitimacy, where tensions among knowledge, trust, and power shape both counter-authoritative narratives and the conditions under which scientific authority itself is produced and sustained.

In this context, conspiratorial epistemology can be understood as emerging from a dialectical tension between epistemic mistrust and biased information processing [51]. The former is not simply a cognitive distortion but is often rooted in epistemic injustice - the systematic exclusion of certain groups from recognition as credible knowers [52]. When grounded in experiences of exclusion or institutional failure, such mistrust may be epistemically justifiable [50], functioning as a form of situated common sense that responds to structural inequities in credibility. It can motivate the search for alternative explanatory frameworks, a process with the potential to challenge dominant epistemic hierarchies.

However, in fragmented and low-credibility information environments—particularly under post-truth conditions—this search becomes increasingly vulnerable to distortion and misinformation. When trustworthy epistemic resources are inaccessible, individuals may default to unreliable accounts as compensatory meaning-making strategies. Limited source-evaluation ability [53], together with motivated reasoning and identity-protective cognition, further amplifies susceptibility to misleading narratives [54]. Over time, these dynamics can reconstruct epistemic mistrust, transforming it from scepticism toward specific content into broader institutional mistrust - casting doubt on science governance as a legitimate source of epistemic authority.

This reconstructed epistemic mistrust is frequently filtered through conspiracy narratives, when instrumentalized, transform scepticism into indiscriminate institutional mistrust. While experiences of injustice can render such mistrust understandable, they do not automatically legitimize the claims built upon it. Conspiracy theories are “for losers” [55], as they often emerge among individuals who feel politically marginalized or powerless, channeling frustration toward perceived out-groups [56] - particularly when access to reliable information or cognitive resources is limited. These narratives are frequently amplified by strategic actors who exploit mistrust to undermine epistemic authority, often without substantive justification [57]. Science-related populism exemplifies this dynamic, valorizing everyday common sense while portraying scientific expertise as detached, elitist, or untrustworthy [58]. This epistemic antagonism is consistently associated with declining confidence in science, even when controlling for education, demographics, and religiosity [59].

Investigating this contested epistemic space confronts a dilemma: the Scylla of equating epistemic dominance with objectivity - thus naturalizing existing credibility hierarchies - and the Charybdis of treating claims from marginalized groups as inherently legitimate. Both positions obscure the relational and contextual processes through which epistemic claims gain credibility. A more robust standard for epistemic justification lies not in presumed objectivity or authenticity, but in whether knowledge practices broaden the social and institutional conditions for inclusive, reflexive, and progressive inquiry.

Accordingly, it is important to distinguish between critical epistemic mistrust - grounded in reflective engagement with scientific authority - and the dogmatic embrace of counter-narratives. Collapsing the public’s diverse modes of engaging with science into simple pro-anti categories obscures the complexity of how people position themselves toward scientific institutions. Orientations toward science develop through the interaction of cultural values, reasoning styles, and institutional trust [20], which together shape responses that are multidimensional and domain-specific [60]. As Godin and Gingras [61] note, *scientific culture* comprises both individual and social dimensions - it involves personal knowledge and attitudes but also the cultural and institutional contexts that confer meaning upon science. Conspiratorial reasoning, therefore, can express either critical questioning or closed-minded rejection, depending on its epistemic grounding and social context.

### C. Reassessing the knowledge–conspiracy–trust pathway: Beyond the deficit model and cognitive polyphasia

Although the multidimensional structure of public understanding of science has been widely examined [22,60,62], less attention has been given to the mediating pathways linking its components. Recent studies have begun to address this gap by identifying indirect relations through which education, values, and cognitive factors interact with scientific trust. Research indicates that education shapes conspiratorial thinking and trust in science through cognitive and value-based mediators [63,64], and that generalized faith in science mediates the effects of religious orthodoxy and conspiracy beliefs on mistrust [65]. Similarly, right-wing authoritarianism predicts pseudoscientific and paranormal beliefs indirectly through social axioms [66], while beliefs about scientific authority and the roles of scientists and citizens are mediated by epistemic trust and deference to expertise [67]. In the health domain, vaccine conspiracy beliefs mediate the relationship between scientific knowledge and vaccine hesitancy, operating through reduced trust in medical science [68,69].

A key limitation in existing research on mediating pathways is the tendency to conflate science-related conspiracism with either institutional mistrust or epistemic deficit, treating them as interchangeable indicators of disengagement from science. This study differentiates these orientations by defining science knowledge as a measure of epistemic competence, conspiracism as an epistemic orientation - a distinct way of constructing and legitimizing knowledge- and institutional mistrust as an attitudinal stance toward scientific authority. Although these dimensions frequently correlate [66,70], they interact in complex ways that warrant closer conceptual separation.

Conspiratorial reasoning operates as an epistemic framework for interpreting uncertainty and perceived injustice through assumptions of hidden intent and systemic deception, while institutional mistrust reflects the evaluative stance arising from this filtered view of scientific authority. Conspiracists often frame their pursuit as a rational, truth-seeking endeavor, positioning themselves as investigators uncovering hidden realities through critical inquiry [35]. As a mediating heuristic, it fills gaps in factual understanding, expressing both a desire for coherence and the constraints of epistemic vulnerability in complex information environments [71]. In this capacity, conspiratorial reasoning can act either as a form of epistemic agency or as a source of distortion, reinterpreting evidence according to alternative criteria of legitimacy when epistemic trust is strained. It is thus neither the inverse of scientific literacy nor a mere expression of institutional mistrust, but a distinct epistemic mode emerging from epistemic mistrust under conditions of uncertainty, positioned along a continuum between reflective engagement and dogmatic closure [72].

Adding to this complexity, the boundary between factual knowledge and evaluative preconceptions is often blurred, a challenge reflected in how the construct is operationalized. Standardized measures of scientific knowledge in surveys frequently lack theoretical grounding and psychometric clarity, showing low discriminatory power, modest reliability, and pronounced ceiling effects, while remaining vulnerable to acquiescence bias and statistical artifacts [13]. These instruments tend to conflate epistemic, attitudinal, and cultural dimensions, interpreting variations in trust or interpretation as knowledge deficits. Many items are culturally inflected, capturing moral and institutional judgments as much as cognitive understanding—in effect, measuring perceptions of authority and institutional mistrust rather than knowledge alone [73]. The result is an implicit fuzziness masked by explicit linearity, as complex epistemic distinctions collapse into a single continuum of correctness.

A similar pattern characterizes measures of science attitudes. Classical models distinguish between perceptions of science’s benefits and reserves about its societal consequences [18,62]. More recent approaches build on this distinction by further differentiating views of science as a driver of technological progress from its role as a moral or institutional authority [59,74]. Rather than existing on a single continuum, these dimensions are analytically orthogonal, delineating a two-dimensional landscape in which people can support scientific progress while expressing reservations about its ethical or institutional impact.

Beyond their multidimensionality and fuzziness, science-related constructs are also embedded in macro influences. Broader contexts shape how science and its institutions are interpreted, extending beyond factual knowledge alone [75]. According to research, the knowledge-attitude relationship varies across national settings - stronger in intermediate economies and weaker in less developed or post-industrial contexts - reflecting how different configurations activate distinct predictors of attitudes [76–78]. However, multilevel evidence is mixed: while regional and national socioeconomic factors account for substantial variation beyond individual knowledge and attitudes in the EU [79], broader cross-national analyses find more limited cultural differences [15]. Regarding conspiracy beliefs, they are shaped by both individual (e.g., scientific knowledge) and contextual factors (e.g., national affluence or regime type) shape, with education and vaccine attitudes exerting stronger effects in wealthier democracies [80], where even those with limited knowledge or higher mistrust of scientists are less prone to endorse science-related conspiracy theories than their counterparts in more authoritarian settings [81]. Overall, higher average knowledge or educational investment appears associated with *context-dependent, non-linear dynamics* that interweave multiple constructs rather than yielding uniform improvements in trust [82].

In conclusion, the relationship between knowledge, conspiratorial reasoning, and science mistrust is best understood as a context-dependent configuration in which individual epistemic and motivational factors are embedded within broader sociocultural conditions. Within this configuration, conspiratorial reasoning may function as one pathway through which knowledge relates to institutional mistrust across levels of analysis.

### D. Hypotheses and model overview

Building on this framework, this study adopts a middle-range theoretical approach [13,83] that integrates these conceptual insights into an empirical multilevel structural model. This enables us to move beyond Deficit and Contextualist paradigms by examining how science knowledge and related covariates are associated with institutional mistrust indirectly via conspiratorial reasoning, and how this pathway is contingent on informational, motivational, and sociocultural conditions.

Scientific and conspiratorial epistemologies represent distinct but interconnected expressions of the same underlying epistemic domain. Contestations of scientific authority are further differentiated into two interrelated components: conspiratorial reasoning, an *epistemic* orientation, and institutional mistrust, an *attitudinal* stance. The former spans a continuum from flexible, case-specific questioning to rigid, generalized suspicion, reflecting differences in how individuals construct and evaluate knowledge claims. The latter, by contrast, captured through the *reserve construct*, reflects a broader normative attitude against science encompassing concerns about institutional trust, epistemic authority, and the societal role of science.

Positioned at the intersection of epistemic and attitudinal domains, conspiracy reasoning operates as a bridge between how individuals *know* and how they *trust*. This intermediary position justifies its specification as a mediating mechanism linking epistemic orientations to attitudinal outcomes. Modelling conspiratorial reasoning as the antecedent of mistrust is consistent with longitudinal evidence showing that counter-authoritative beliefs more reliably predict subsequent declines in trust in experts, institutions, and science-related behaviours than the reverse [84]. The model further incorporates three clusters of covariates - Cognitive and Informational Factors, Perceptual and Science-Related Beliefs, and Ideological and Sociocultural Factors - to account for additional sources of variation influencing both conspiracy beliefs and mistrust.

***H1.** The indirect effect of science knowledge on mistrust through conspiratorial reasoning will be significant and, relative to the covariates included in the model, comparatively stronger than the corresponding indirect effects.*

Beyond the core mediational pathway, and consistent with our contingency framework, the model incorporates moderated mediation and mediated moderation, in which knowledge × covariate interactions shape and transmit effects on mistrust through conspiratorial reasoning.

***H2a.** The indirect association between science knowledge and mistrust via conspiratorial reasoning will be conditioned by informational, perceptual, ideological, and sociocultural variables.*

While individual epistemic capacities establish a baseline for the relationship between knowledge and attitudes, their effects are shaped by broader cultural and political contexts [12,61]. Religiosity, in particular, represents a salient contextual factor influencing how scientific knowledge relates to mistrust through conspiratorial reasoning. The literature remains divided: some studies emphasize incompatibilities between religious and scientific epistemologies—especially concerning claims to epistemic authority [19,85,86] - whereas others find that religious commitment and scientific understanding can coexist within individuals and communities [41]. Building on this mixed evidence, we propose that the conspiracy-mediated link between scientific knowledge and mistrust is contingent on the broader religious climate that defines epistemic legitimacy and evaluative norms, reflecting neither a universal deficit nor a coexistence pattern.

***H2b.*** *The mediated pathway between individual knowledge and mistrust will be conditioned by macro-level religiosity, such that its strength and direction depend on the prevailing religious climate.*

At the macro level, the model extends the individual-level mediation framework to test whether similar relationships emerge among aggregated constructs, without committing ecological fallacy. Specifically, it examines whether regions with higher average science knowledge show lower prevalence of conspiratorial orientations and, in turn, reduced mistrust. This captures how collective scientific understanding and shared epistemic climates reflect broader cognitive-informational, political-cultural, and socioeconomic conditions shaping societal orientations toward science and authority.

***H3.** At the macro level, the indirect effect of science knowledge on mistrust through conspiratorial reasoning will be significant and expected to exceed the corresponding indirect effects of the covariates included in the model.*

## III. Data and methods

### A. Data

Data were drawn from Eurobarometer 95.2 (ZA7782), a cross-national survey conducted in April–May 2021 across 38 European countries. The target population comprised residents aged 15 and older, selected via multistage, stratified random sampling by region and population size. Interviews were administered using computer-assisted personal (57%) and web interviewing (43%) modes. The initial dataset included 37,079 individuals in 308 regions. After excluding cases with >20% missing attitude or >30% missing knowledge responses, the final analytic sample comprised 35,075 participants.

Missing data in observed covariates were addressed using random-forest imputation (**missRanger**) with auxiliary covariates representing demographics, political orientation, and paradata. to support the plausibility of a **MAR** mechanism. For the knowledge battery, “Don’t know” responses were coded as incorrect (0/False) prior to MIRT estimation as explained below, whereas missingness in other item batteries for latent constructs was retained as missing (typically <4%) and handled within the measurement stage without item-level imputation. MIRT-derived factor scores were then used as observed inputs in the multilevel structural models; because pooling multilevel SEM estimates across multiple imputations is nontrivial in this setting, models were estimated on the resulting single completed dataset.

### B. Measures

#### Latent variables.

All latent constructs were estimated using multidimensional item response theory (MIRT). For dichotomous and graded (Likert-type) indicators, MIRT provides item parameters (difficulty/thresholds and discrimination) and EAP factor-score estimates with associated uncertainty (score SEs). These factor scores were then used as observed inputs in the multilevel structural models; factor-score uncertainty was evaluated via sensitivity checks reported in S1 Appendix. This two-stage scores-as-observed approach is pragmatic and transparent, but it does not fully propagate latent-score uncertainty into the structural model and may therefore attenuate some associations, particularly smaller effects. Reliability was generally good across constructs and was modest for some key measures, including knowledge and conspiracy-related factors (see S1 Appendix for details). Because MIRT factor scores were used as observed inputs in subsequent models, effects involving constructs with moderate reliability may be conservatively attenuated, reducing precision and potentially underestimating true associations, and are therefore interpreted with appropriate caution. The full wording of all items used in the analysis is provided in S1 Appendix.

#### Science Knowledge (Knowledge) and Conspiracy Reasoning (Conspiracy).

We derived MIRT-based factor scores from a 10-item battery spanning factual knowledge and science-related conspiratorial content (originally 11, one item was excluded due to unstable estimation). “Don’t know” responses were coded as incorrect (0/False), consistent with common practice in objective knowledge measurement. To ensure stable IRT calibration, respondents with excessive item DK (>70% DK across all items) were excluded from the measurement calibration step. Dimensionality was examined with an exploratory multidimensional IRT model, which supported a three-dimensional representation consistent with **Basic Literacy** (basic school knowledge), **Scientific Knowledge** (mechanistic understanding of physical/biological processes), and **Conspiratorial Reasoning** (endorsement of narratives involving hidden agendas, suppression, or control). We then estimated a confirmatory three-factor 2PL MIRT model reflecting these facets within a broader epistemic domain: conspiratorial reasoning is not conceptualized as the opposite of knowledge, but as a partially overlapping interpretive orientation toward evidence and epistemic authority. The confirmatory model fixed the Knowledge–Conspiracy factor covariance at a modest positive value (0.30) to reflect partial dependence while preserving conceptual distinction. EAP factor scores (and score SEs) were extracted for use as observed inputs in subsequent multilevel structural models.

In the confirmatory specification, two human-evolution items (“earliest human beings coexisted with dinosaurs” and “human species evolved”) were specified to cross-load on both the Scientific Knowledge and Conspiratorial Reasoning factors, consistent with our framework in which factual understanding and epistemic contestation are distinct yet intersecting orientations. Substantively, the conspiratorial factor captures an epistemic orientation ranging from sceptical contestation to more generalized closure toward the epistemic authority of science, such that stronger alignment across conspiracy items reflects broader, dogmatic suspicion rather than a simple knowledge deficit or heuristic scepticism.

Final scores were derived from the confirmatory model; the Basic Literacy factor was excluded from scoring due to low reliability (ω = .46). Reliability for Scientific Knowledge (ω = .54) and Conspiratorial Reasoning (ω = .65) was modest to moderate and sufficient for comparing score variation at the individual and regional levels; remaining uncertainty was evaluated via sensitivity analyses reported in S1 Appendix. At the same time, these reliability estimates indicate non-trivial measurement error, which may attenuate observed associations and reduce the precision of the estimates, and the corresponding findings should therefore be interpreted with caution.

#### Science attitudes—reserve (Mistrust/Reserve).

A 19-item battery assessed science attitudes on a five-point Likert scale and was modelled using a graded MIRT specification. Exploratory MIRT supported three dimensions consistent with prior work: **Reserve** (mistrust/reserve toward science and scientists; 7 items), **Promise** (optimism about science’s benefits; 9 items), and **Deference** (respect for scientific authority; 3 items). Only the **Reserve** dimension was retained as the focal indicator of mistrust/reserve in the structural models, capturing skepticism toward scientific authority, including concerns about scientists’ power, accountability, trustworthiness, and the societal implications of scientific progress. In the final solution, Reserve items showed moderate-to-strong discrimination (e.g., item loadings on the Reserve factor approximately **.50–.75**), supporting a coherent latent construct. Reserve showed good reliability (ω = .78); reliabilities for the other extracted dimensions (Promise, Deference) are reported in S1 Appendix.

#### Science Engagement (Engagement).

A 12-item battery assessed science-related participation on a four-point frequency scale. Exploratory MIRT supported two correlated dimensions: **Active Engagement** (8 items; e.g., petitions/demonstrations, NGO activities, contacting public authorities) and **Passive Engagement** (4 items; e.g., talking about science with family/friends, watching documentaries/reading science- and technology-related publications). Only **Passive Engagement** was retained for the main analyses due to pronounced floor effects in the Active Engagement items and its stronger theoretical relevance to everyday information exposure. The two-factor solution showed strong item discrimination (e.g., Passive loadings up to **.94**) and good reliability for both dimensions (**ω = .83** active; **ω = .81** passive).

#### Negative scientist perceptions (Negative Scientist Perceptions).

This construct was estimated using a unidimensional 2PL MIRT model from 10 binary trait attributions (e.g., narrow-minded, arrogant, immoral). Positive trait items were reverse-coded so that higher scores indicated greater attribution of negative characteristics. The final solution showed moderate-to-strong discrimination (standardized loadings: **.40–.86**; h²: **.16–.75**) and modest reliability (**ω = .58**). As with other modest-reliability constructs, effects involving this construct may be attenuated due to measurement error and should be interpreted with caution.

#### Unequal benefits of science and technology (Unequal Benefits).

This construct was estimated using a graded MIRT model from three five-point Likert items selected iteratively from an initial eight-item battery to achieve a theoretically coherent and well-fitting specification. Items captured perceptions that scientific and technological progress disproportionately benefits privileged groups (e.g., “Science mostly improves the lives of the well-off”). The final three-item solution showed strong discrimination (standardized loadings: **.82–.87**; h²: **.67–.75**) and good reliability (**ω = .77**).

#### Observed variables.

Single-item predictors captured populist decision-making (), religiosity, left–right political orientation, use of social media as a main source of science information (social media), worldview (cooperative vs. threat-oriented), self-perceived information about scientific discoveries (information), and perceived inefficacy of science (perceived vaccine inefficacy; reverse-coded evaluations of vaccines and disease pr **e**xperts vs. people evention). Religiosity was measured on a 10-point scale, whereas the remaining single-item predictors used 4- or 5-point Likert type response scales, with binary indicators for **experts vs people**, **coop vs threat**, and **social media**.

#### Control variables.

Control variables included socio-demographics—age, respondent and parental education, and economic strain (difficulty paying bills)—in line with standard practices in the conspiracy literature [87], as well as paradata [80], such as interview mode (CAPI vs. CAWI), and guessing propensity (ratio of incorrect to incorrect plus DK responses)

#### Level 2 (Regional) variables.

Regional variables included both aggregated individual constructs and external contextual indicators. Regional means were computed automatically within the lavaan MSEM framework for Conspiracy, Mistrust, Knowledge, Unequal Benefits, Negative Scientist Perceptions, Religiosity, and Satisfaction with Democracy.

Regional electoral integrity perceptions were operationalized as a latent construct based on an eight-item, four-point EVS/WVS 2017–2022 battery assessing electoral fairness and institutional integrity, while trust toward people of another nationality (single observed item) was drawn from the same source; both indicators were aggregated to the regional level. In the final between-level specification, the electoral integrity construct was not included due to conceptual overlap with other political-legitimacy indicators and resulting instability when entered jointly, whereas out-group trust was retained as the focal intergroup-attitudinal contextual measure.

Contextual indicators included per capita purchasing power standard (PPS) and the gender employment gap [88], indexing regional economic development and gender inequality. Because the EVS/WVS dataset did not cover Belgium, Ireland, Luxembourg, and Malta, regional scores for Electoral Integrity and Outgroup Trust in these countries were imputed (missRanger, MAR assumed) based on regional covariates (PPS, university enrolment, disposable income, gender employment gap, and high-tech employment).

#### Use of AI tools.

Artificial intelligence tools were used to assist with language editing, stylistic refinement, and support in R coding. These tools served solely as auxiliary aids; all substantive analyses, coding logic, interpretation of results, and theoretical development were independently conducted and verified by the authors.

### C. Analysis

Multilevel structural equation models were estimated in the lavaan package [89], decomposing variance into within- and between-region components.

**Fig 1:**
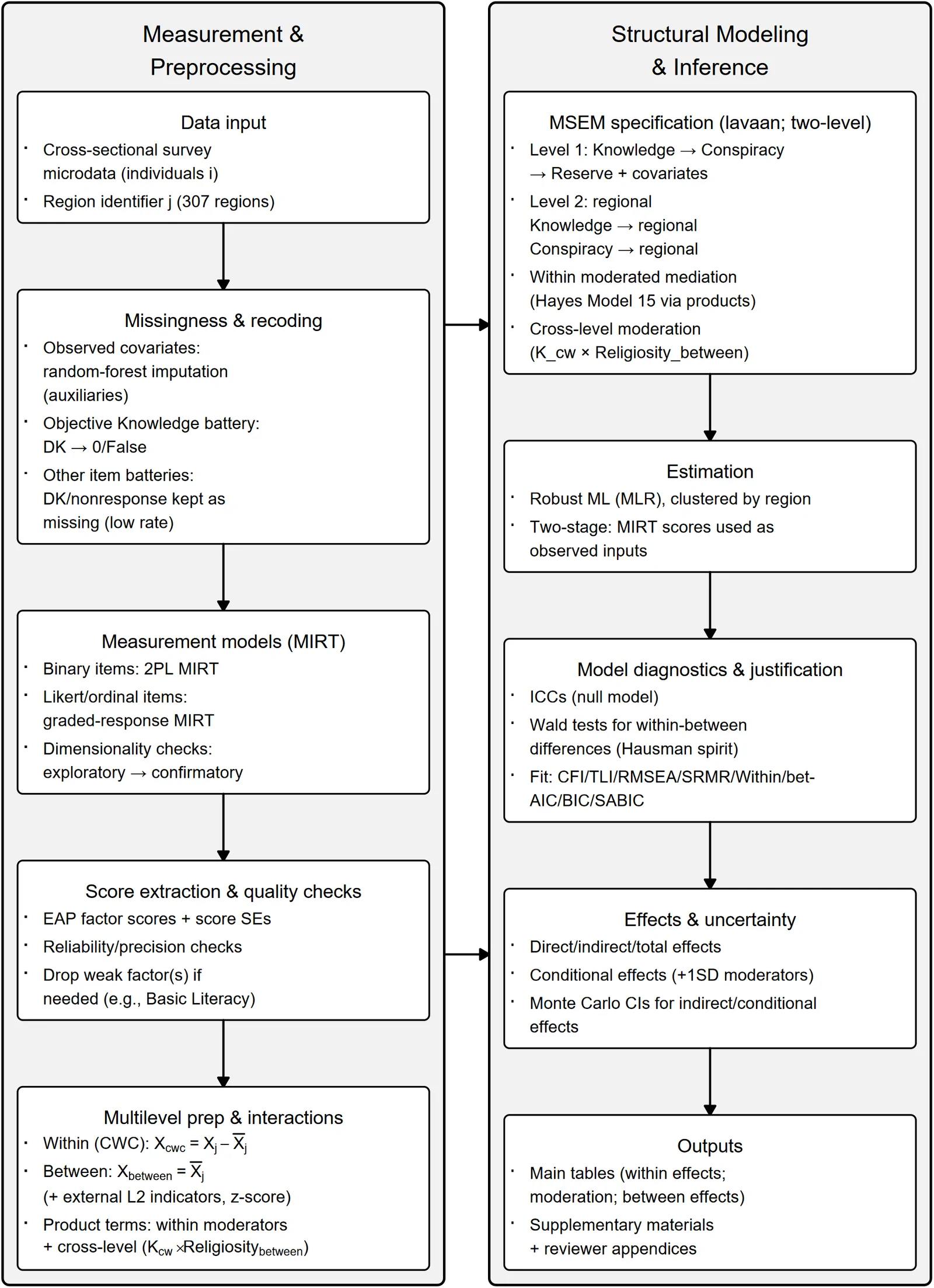
Analytical workflow of the study.

Intraclass correlations indicated meaningful regional clustering (ICC = .27 for Conspiracy; ICC = .22 for Mistrust/Reserve). In a Hausman-like spirit, Wald tests showed significant between effects (a_b ≠ 0: χ²(1)=1092.97, p < .001; c_b ≠ 0: χ²(1)=6.01, p = .014) and significant within–between differences (a_w = a_b, c_w = c_b rejected: χ²(2)=206.80, p < .001), supporting the MSEM decomposition across levels. The analytical workflow of the study is summarized in Fig 1.

#### Model structure.

All multilevel models were estimated at two levels (individual *ii*i nested in region *jj*j). For each score-based variable X, we decomposed it into within- and between-region components using the observed region mean:


$$\begin{array}{c} X_{ij}=X_{𝕚j}^{w}+X_{j}^{B},\;X_{𝕚j}^{w}=X_{ij}-{\overset{―}{X}}_{j},\;X_{j}^{B}={\overset{―}{X}}_{j} \end{array}$$

where $\overset{―}{X_{j}}$ is the region mean. In the dataset, within components are denoted with the suffix “_*cwc*_ ” and between components with “_*between*_ ”.

At Level 1 (within), the mediator (Conspiracy) and outcome (mistrust/reserve) equations are:


$$\begin{array}{c} Conspiracy\_cwc_{ij}=\text{\textasciitilde{}}\alpha_{kn}Knowledge\_cwc_{ij}+Z_{𝕚j}^{WT}\alpha +u_{\dot{i}j}^{\left(C\right)}, \end{array}$$

$$\begin{array}{c} eserve\_cwc_{ij}=bConspiracy\_cwc_{ij}+c_{kn}Knowledge\_cwc_{ij}+Z_{𝕚j}^{WT}\gamma +u_{ij}^{\left(R\right)} \end{array}$$

where $Z_{𝕚j}^{wT}$ includes the full set of within-level covariates and moderators used in the lavaan specification (entered directly and via precomputed product terms).

At Level 2 (between), we specified:


$$\begin{array}{c} Conspiracy\_between_{j}=\text{\textasciitilde{}}a_{kN}^{B}Knowledge\_between_{j}+Z_{j}^{BT}\alpha^{B}+u_{j}^{\left(C\right)}, \end{array}$$

$$\begin{array}{c} eserve\_between_{j}=b^{B}Conspiracy\_between_{j}+c_{kn}^{B}Knowledge\_between_{j}+Z_{j}^{BT}\gamma^{B}+u_{j}^{\left(R\right)} \end{array}$$

With $Z_{j}^{BT}$ denoting region-level contextual predictors.

Indirect and total effects were defined as:


$$\begin{array}{c} ind_{kn}=a_{kn}\cdot b,\;total_{kn}=c_{kn}+ind_{kn},\;ind\_kn\_b\;=a_{kn}^{B}\cdot b^{b},\;total\_kn\_b\;=c_{kn}^{B}+ind\_kn\_b \end{array}$$

Moderation was modeled using centred product terms (e.g., Knowledge_cwc×Moderator _cwc and Conspiracy cwc ×Moderator cwc on Level 1), and cross-level moderation was represented by interactions between within-level knowledge and between-level regional context.

#### Model estimation.

Models were estimated using maximum likelihood with the NLMINB optimizer. Indirect effects were evaluated using Monte Carlo confidence intervals to account for potential non-normality. Because lavaan does not support random slopes, slopes were constrained to be equal across regions. Model fit was evaluated using standard fit indices [90], indicating good overall fit (CFI = .955, RMSEA = .029, SRMR_within_ =.013, SRMR_between_ = .008), although the TLI was comparatively lower (TLI = .875), which is expected in complex multilevel specifications with many predictors and constraints where incremental fit indices can be sensitive to model complexity. At the within level, the model explained 29.1% of the variance in Conspiracy and 32.2% of the variance in Reserve. At the between level, the corresponding explained variances were 83.1% and 53.3%, respectively.

Interaction terms were computed externally from centered variables. Level-1 predictors were group-mean centered to isolate within-region effects, whereas the corresponding regional means were grand-mean centered to represent between-region effects. All Level-2 contextual variables were z-standardized. Three observed predictors—social media, experts vs people and coop vs threat—were binary and were entered in the MSEM as 0/1 indicator variables. Several additional observed covariates were measured on Likert-type scales (including 4-point and 10-point formats) and were treated as approximately continuous in the structural model. Although all covariates described in the Measures section were initially considered at their respective levels, two Level-2 predictors were ultimately removed from the final model: (i) regional unequal benefits of science, because its inclusion produced strong suppression/overlap due to shared between-level variance among unequal benefits, conspiracy climate, and mistrust/reserve (substantially attenuating the Conspiracy_between → Reserve_between pathway), and (ii) an electoral–democratic integrity index, excluded for parsimony due to conceptual redundancy with other governance indicators and instability when entered jointly.

As a robustness check, we applied Benjamini–Hochberg/FDR adjustments across families of tests (within-level regressions, between-level regressions, and defined/indirect effects). The main conclusions (including the focal Knowledge → Conspiracy → mistrust/reserve pathway at both within and between levels) remained statistically robust; a small subset of secondary interaction/index terms and some contextual covariates were attenuated and should be interpreted cautiously as exploratory.

## IV. Results

### A. Descriptive statistics and correlations

Tables 1 and 2 present means, standard deviations, and zero-order correlations for individual-level (Level 1) and regional-level (Level 2) variables. At Level 1, scientific knowledge was moderately negatively correlated with conspiracy beliefs (r = −.50) and mistrust/reserve (r = −.28), while conspiracy beliefs were positively associated with mistrust/reserve (r = .34). A similar pattern appeared at Level 2: regional average knowledge showed strong negative correlations with both regional conspiracy climate (r = −.85) and regional mistrust/reserve (r = −.64), and regional conspiracy was strongly correlated with regional mistrust/reserve (r = .68). Correlations among other variables at both levels ranged from weak to moderate.

**Table 1:** Means, standard deviations, and zero-order correlations (individual level, within-region components).

Variable	M	SD	1	2	3	4	5	6	7	8	9	10	11	12	13	14
1. Conspiracy	−0.003	0.819	1.00													
2. Reserve (mistrust)	−0.021	1.240	0.34	1.00												
3. Science Knowledge	0.001	0.756	−0.50	−0.28	1.00											
4. Passive Engagement	0.091	1.190	−0.17	−0.21	0.26	1.00										
5. Info on Scientific Discoveries	1.800	0.632	−0.11	−0.13	0.17	0.40	1.00									
6. Unequal Benefits	0.015	0.890	0.13	0.35	−0.05	0.02	−0.01	1.00								
7. Democracy Satisfaction	2.560	0.845	−0.17	−0.11	0.03	0.05	0.08	−0.10	1.00							
8. Cooperation vs. Threat	0.310	0.463	0.16	0.18	−0.11	−0.10	−0.05	0.03	−0.09	1.00						
9. Religiosity	5.170	2.810	0.14	0.22	−0.16	−0.07	−0.07	0.04	0.03	0.05	1.00					
10. Left–Right Orientation	2.940	1.050	0.07	0.06	−0.05	−0.04	0.04	−0.08	0.05	0.11	0.12	1.00				
11. Experts vs. People	0.246	0.432	0.15	0.18	−0.09	−0.06	−0.02	0.03	−0.06	0.14	0.05	0.04	1.00			
12. Negative Scientist Perceptions	0.000	0.820	0.21	0.34	−0.08	−0.07	−0.07	0.14	−0.16	0.14	0.06	0.06	0.18	1.00		
13. Vaccine Inefficacy	1.650	0.788	0.20	0.14	−0.08	−0.08	−0.05	0.00	−0.14	0.09	0.05	0.02	0.15	0.24	1.00	
14. Social Media Use	0.296	0.456	0.04	−0.02	0.01	0.05	0.06	0.03	−0.03	−0.02	−0.06	0.00	0.00	0.00	0.04	1.00

**Note.** Entries are Pearson zero-order correlations computed on within-region (cluster-mean centered) components (Level 1). Means (M) and standard deviations (SD) are reported for each variable. Correlations are reported for descriptive purposes only. No evidence of problematic multicollinearity was observed (all |r| < .70).

**Table 2:** Means, standard deviations, and zero-order correlations (regional level).

Variable	M	SD	1	2	3	4	5	6	7	8	9	10	11	12	13
1. Conspiracy beliefs	0.06	0.44	1.00												
2. Reserve (mistrust)	0.08	0.59	0.68	1.00											
3. Science knowledge	−0.06	0.38	−0.85	−0.64	1.00										
4. Unequal benefits of science	0.00	0.35	0.31	0.62	−0.23	1.00									
5. Democracy satisfaction	0.00	0.38	−0.57	−0.42	0.42	−0.33	1.00								
6. Democracy(electoral)trust (z)	−0.06	0.93	−0.68	−0.48	0.63	−0.10	0.43	1.00							
7. Religiosity	0.00	1.36	0.61	0.56	−0.61	0.23	−0.23	−0.51	1.00						
8. Cooperation vs. threat worldview	0.00	0.15	0.36	0.37	−0.27	0.07	−0.23	−0.32	0.31	1.00					
9. Trust in other nations (z)	−0.18	1.03	−0.68	−0.49	0.56	−0.21	0.44	0.53	−0.56	−0.30	1.00				
10. Purchasing power (z)	−0.16	0.87	−0.65	−0.52	0.62	−0.23	0.48	0.62	−0.44	−0.30	0.54	1.00			
11. Gender employment inequality (z)	0.19	1.21	0.51	0.37	−0.47	0.28	−0.36	−0.46	0.57	0.25	−0.54	−0.42	1.00		
12. Negative scientist perceptions	0.00	0.33	−0.15	−0.03	0.30	−0.18	0.13	0.05	−0.09	0.28	0.11	0.15	−0.24	1.00	
13. Experts vs. people orientation	0.00	0.14	0.31	0.40	−0.23	0.05	−0.05	−0.26	0.27	0.54	−0.16	−0.19	0.17	0.45	1.00

**Note.** Entries are Pearson correlations computed on region-level aggregates (Level 2; **J = 307** regions). Means (M) and standard deviations (SD) describe the distributions of regional aggregates. Correlations are reported for descriptive purposes. Variable labels omit the “_between” suffix for readability.

At the regional level, perceived unequal benefits of science was strongly correlated with mistrust/reserve (r = .62) and moderately correlated with conspiracy (r = .31), consistent with the overlap/suppression pattern observed when this predictor was added to the between-level structural model (attenuating the Conspiracy__between_ → Reserve__between_ pathway). These patterns suggest that some Level-2 constructs are closely intertwined, limiting the extent to which distinct regional mechanisms can be cleanly separated within a single model; this issue is discussed further below.In addition, democratic trust (electoral integrity) is reported descriptively (Table 2) but was not retained in the final between-level specification for parsimony given its overlap with other governance-related indicators.

### B. Within level (Individual) effects

#### Main mediation path: Knowledge → conspiracy → mistrust.

Scientific knowledge was strongly and negatively associated with conspiracy beliefs (β = −.41, p < .001). Conspiracy beliefs, in turn, were positively associated with mistrust/reserve (β = .142, p < .001). The direct effect of knowledge on mistrust was weaker (*β* = −.10, *p* < .001) than the corresponding bivariate correlation (*r* = −.39), consistent with prior research showing attenuation once confounding variables are controlled (see Table 3). Knowledge exerted a significant indirect effect on mistrust via conspiracy beliefs (β = −.095, 95% CI [−.103, −.088]), yielding a total effect of β = −.260, 95% CI [−.276, −.245] (see Table 3). These findings support the hypothesized mediating role of conspiracy beliefs in the relationship between scientific knowledge and mistrust/reserve at the individual level, net of the covariates included in the model.

**Table 3:** Standardized path coefficients (β), monte carlo indirect effects, and total effects.

Predictor	Path a (→ Conspiracy Beliefs) β (SE)	Path c′ (→ Mistrust-Reserve) β (SE)	Indirect Effect (a × b) [95% CI]	Total Effect [95% CI]
**Conspiracy (Mediator)**	—	0.142 (0.008)*	—	—
**Cognitive and Informational Factors**				
Science Knowledge	−0.414 (0.005)*	−0.102 (0.009)*	**−0.095** [−0.103, −0.088]	**−0.260** [−0.276, −0.245]
Passive Science Engagement	**−0.016 (0.003)** **	−0.111 (0.005)*	**−0.002** [−0.004, −0.001]	**−0.116** [−0.126, −0.105]
Info on Scientific Discoveries	**0.012 (0.006)** *	**−0.011 (0.009)** *	**0.003** [0.001, 0.006]	**−0.016** [−0.033, 0.001]
Social Media Use	0.040 (0.008)*	**−0.006 (0.011)**	**0.014** [0.010, 0.017]	**−0.002** [−0.024, 0.020]
**Perceptual and Science-Related Beliefs**				
Unequal Benefits of Science	0.089 (0.004)*	0.287 (0.006)*	**0.016** [0.014, 0.018]	**0.381** [0.369, 0.392]
Negative Scientist Characteristics	0.097 (0.004)*	0.207 (0.007)*	**0.019** [0.016, 0.021]	**0.300** [0.287, 0.313]
Perceived Vaccine Inefficacy	0.110 (0.005)*	**0.014 (0.007)** **	**0.023** [0.020, 0.025]	**0.042** [0.029, 0.056]
**Ideological and Sociocultural Factors**				
Religiosity	0.049 (0.001)*	0.123 (0.002)*	**0.003** [0.002, 0.004]	**0.056** [0.052, 0.060]
Right-Leaning Orientation	0.040 (0.003)*	0.023 (0.005)*	**0.006** [0.005, 0.007]	**0.030** [0.021, 0.040]
Democracy Satisfaction	−0.106 (0.004)*	**0.001 (0.006)**	**−0.021** [−0.023, −0.018]	**−0.020** [−0.032, −0.007]
Threat-Oriented Worldview	0.056 (0.007)*	0.063 (0.011)*	**0.019** [0.015, 0.022]	**0.168** [0.146, 0.189]
Populist Decision Preference	0.059 (0.008)*	0.073 (0.012)*	**0.021** [0.018, 0.025]	**0.205** [0.182, 0.228]
**Controls**				
Age	**−0.002 (0.002)**	0.057 (0.003)*	—	—
Education Level	**−0.010 (0.004)** *	−0.036 (0.006)*	—	—
Mother’s Education	−0.022 (0.004)*	**−0.010 (0.006)**	—	—
Father’s Education	**−0.003 (0.004)**	**−0.004 (0.006)**	—	—
Payment Difficulty (Economic Strain)	0.043 (0.006)*	**0.012 (0.009)** *	—	—
Guessing Propensity	**0.009 (0.006)** †	**−0.011 (0.010)** **	—	—
Explained Variance (R²)	0.291	0.322		

**Note.** Coefficients are fully standardized (Std.all) estimates, with standard errors from the corresponding Std.Err column in parentheses. Confidence intervals for indirect and total effects are Monte Carlo 95% intervals. ***p < .001, **p < .01, *p < .05, †p ≈ .05.

#### Cognitive and informational variables.

Passive science engagement showed a small negative association with conspiracy beliefs and a stronger negative direct association with mistrust/reserve, producing an overall negative total effect (Table 3). Self-perceived information about scientific discoveries showed positive association with conspiracy beliefs and a negative direct effect on mistrust/reserve, resulting in a near-zero total effect once indirect and direct pathways were combined. Social media use was positively related to conspiracy beliefs and affected mistrust/reserve primarily through an indirect pathway via conspiracy beliefs, while its total effect was negligible (Table 3).

#### Perceptual and science-related predictors.

Perceived unequal benefits of science and negative views of scientists were among the strongest predictors of mistrust/reserve. Both were positively associated with conspiracy beliefs and showed strong direct effects on mistrust/reserve, with smaller positive indirect effects via conspiracy beliefs. Consequently, both produced large total effects (Unequal benefits total β = .381, 95% CI [.369, .392]; Negative scientist perceptions total β = .300, 95% CI [.287, .313]; Table 3). Perceived vaccine inefficacy was positively associated with conspiracy beliefs and showed a small positive total effect on mistrust/reserve after accounting for indirect pathways (Table 3).

#### Ideological and sociocultural factors.

Religiosity and right-leaning orientation showed small but consistent positive indirect effects via conspiracy beliefs and positive total effects on mistrust/reserve (Table 3). Satisfaction with democracy was negatively associated with conspiracy beliefs and predicted mistrust/reserve primarily through its indirect pathway (Table 3). Threat-oriented worldview and populist decision preference demonstrated partial mediation: both contributed to mistrust/reserve through conspiracy beliefs and also retained positive direct associations, resulting in sizeable total effects (Table 3).

#### Knowledge-conditioned moderated mediation.

Moderated mediation models (Hayes, Model 15) indicated that passive science engagement strengthened the indirect effect of knowledge on mistrust/reserve through conspiracy beliefs (index = −.022, 95% CI [−.029, −.015]; Table 4). Conditional estimates showed the indirect effect was more negative at high engagement (High = −.118 [−.129, −.108]) than low engagement (Low = −.072 [−.082, −.062]), with corresponding differences in the total effect (High = −.321 [−.343, −.298] vs Low = −.200 [−.222, −.178]). Self-perceived information about scientific discoveries also showed a smaller but significant moderated mediation effect (index = −.013, 95% CI [−.025, −.001]). Religiosity moderated the mediation in the opposite direction (index = .005, 95% CI [.002, .007]), consistent with attenuation of the negative indirect effect at higher religiosity. In contrast, perceived unequal benefits of science did not exhibit reliable moderated mediation in the revised model (index = −.003, 95% CI [−.011, .005]).

**Table 4:** Knowledge-conditioned interaction effects (mediated moderation and moderated mediation—hayes model 15).

*Moderated mediation (Hayes Model 15)*				
Moderator	Interaction Coefficients (β)	Indirect Pathway	Conditional Indirect Effect (Low / High) [95% CI]	Conditional Total Effect (Low / High) [95% CI]
Passive Science Engagement	**K×Eng:** −.023***; **C×Eng:** .035***	**Index = −.022** [−.029, −.015]	**Low:** −.072 [−.082, −.062] / **High:** −.118 [−.129, −.108]	**Low:** −.200 [−.222, −.178] / **High:** −.321 [−.343, −.298]
Self-Perceived Information	**K×Info:** −.010†; **C×Info:** .012*	**Index = −.013** [−.025, −.001]	**Low:** −.087 [−.098, −.077] / **High:** −.103 [−.113, −.093]	**Low:** −.236 [−.258, −.214] / **High:** −.285 [−.307, −.263]
Religiosity	**K×Rel:** .010†; **C×Rel:** −.018***	**Index = .005** [.002, .007]	**Low:** −.107 [−.117, −.097] / **High:** −.083 [−.093, −.074]	**Low:** −.287 [−.308, −.266] / **High:** −.233 [−.254, −.213]
Unequal Benefits of Science	**K×UB:** −.016**; **C×UB:** .004 (ns)	**Index = −.003** [−.011, .005]	**Low:** −.092 [−.103, −.082] / **High:** −.098 [−.108, −.088]	**Low:** −.232 [−.253, −.210] / **High:** −.289 [−.310, −.268]
***Mediated moderation***				
**Moderator**	**Interaction Coefficients (β)**	**Indirect Pathway**	**Conditional / Mediated Effect [95% CI]**	**Conditional Total Effect [95% CI]**
Negative Scientist Characteristics (K×NegSci)	**Conspiracy (a_kno_neg):** −.001 (ns); **Mistrust (c_kno_neg):** .026***	**ab = 0.000 [−.003, .002]**	**Indirect:** 0.000 [−.003, .002]	**Total:** 0.052 [.034, .070]
Regional Religiosity (Level 2) (cross-level K×Rel_between)	**Conspiracy (a_kn_cross):** −.012**; **Mistrust (c_kn_cross):** .037***	**Index = −.002 [−.004, −.001]**	**Low:** −.092 [−.100, −.085] / **High:** −.098 [−.106, −.090]	**Low:** −.320 [−.341, −.299] / **High:** −.201 [−.223, −.179]

**Note.** “Low/High” conditional effects are evaluated at **±1 SD** of the moderator. CIs are Monte Carlo 95% intervals. Significance stars reflect the underlying interaction coefficients’ p-values where provided; for index terms, significance is reflected by whether the CI excludes 0. ***p < .001, **p < .01, *p < .05, †p < .10.

#### Mediated moderation.

Mediated moderation via the Knowledge × negative scientist perceptions interaction was not supported (indirect effect via conspiracy beliefs approximately zero; Table 4). However, the interaction retained a positive total association with mistrust/reserve (β = .052, 95% CI [.034, .070]), suggesting a primarily direct (non-mediated) interaction effect.

#### Cross-level moderation.

Cross-level moderated mediation indicated a small but non-zero variation in the mediated pathway as a function of regional religiosity (index = −.002, 95% CI [−.004, −.001]; Table 4). Conditional estimates suggested modest differences in indirect effects across low vs high regional religiosity, while total effects differed more noticeably across contexts (Table 4), indicating that cross-level moderation operated primarily through the direct component in the revised model.

### C. Between-level (Regional) effects

At the regional level, knowledge was strongly and negatively associated with conspiracy beliefs (β = −.616, p < .001) and also negatively associated with mistrust/reserve (β = −.204, p = .016). This produced a sizable indirect effect via regional conspiracy beliefs (−.265, 95% CI [−.447, −.088]) and a large total effect (−.579, 95% CI [−.765, −.392]), indicating that regions with higher knowledge levels exhibited lower overall mistrust/reserve (see Table 5).

**Table 5:** Between-level (regional) mediation.

Between-Level (Regional) Mediation Effects via Conspiracy Beliefs				
Predictor	Path a → Conspiracy Beliefs (β, SE)	Path c′ → Mistrust (β, SE)	Indirect Effect (a × b) [95% CI]	Total Effect [95% CI]
**Conspiracy Beliefs (Mediator)**	—	**0.279 (0.127)** **	—	—
**Cognitive & Perceptual Contexts**				
Science Knowledge	−0.616 (0.043)*	**−0.204 (0.131)** *	**−0.265** [−0.447, −0.088]	**−0.579** [−0.765, −0.392]
Negative Scientist Perceptions	**0.081 (0.037)** **	**0.071 (0.082)**	**0.040** [0.008, 0.084]	**0.164** [0.004, 0.329]
**Political & Ideological Contexts**				
Democracy Satisfaction	−0.195 (0.033)*	**−0.101 (0.079)** *	**−0.085** [−0.152, −0.027]	**−0.242** [−0.389, −0.097]
Religiosity	**0.060 (0.011)** †	0.232 (0.025)*	**0.007** [−0.001, 0.019]	**0.107** [0.060, 0.156]
Threat-Oriented Worldview	**0.040 (0.079)**	**0.100 (0.176)** *	**0.043** [−0.015, 0.120]	**0.426** [0.070, 0.779]
Out-Group Distrust	−0.174 (0.014)*	**0.016 (0.033)**	**−0.028** [−0.051, −0.008]	**−0.019** [−0.082, 0.044]
**Economic Contexts**				
Purchasing Power	**−0.055 (0.016)** †	**−0.074 (0.037)**	**−0.010** [−0.027, 0.002]	**−0.061** [−0.133, 0.012]
Gender Employment Inequality	**0.004 (0.011)**	**−0.064 (0.025)**	**0.001** [−0.009, 0.010]	**−0.031** [−0.081, 0.020]
Explained Variance (R²)	0.831	0.533		

**Note.** β values are **Std.all** from the lavaan output; SEs are the corresponding **Std.Err**. Monte Carlo 95% CIs are reported for indirect and total effects. ***p < .001, **p < .01, *p < .05, †p < .10.

Given the very strong zero-order association between regional knowledge and conspiracy climate, however, this pathway should be interpreted with some caution, as these constructs may be difficult to disentangle fully at Level 2. This strong association may partly reflect the fact that both constructs capture related epistemic dimensions of science-related orientations at the regional level, even though they are not theoretically identical

It is crucial to note that the broader (pre-specified) between-level model initially included regional perceived unequal benefits of science. However, in sensitivity analyses, adding unequal benefits led the Conspiracy_between → reserve_between path to attenuate to near zero, while unequal benefits remained a strong predictor of reserve/mistrust. This pattern is consistent with substantial shared between-level variance among unequal benefits, conspiracy climate, and reserve/mistrust (i.e., an overlap/suppression effect), suggesting that unequal benefits captures a closely related contextual “institutional injustice” pathway rather than a distinct component of the conspiracy-mediated mechanism. Accordingly, we present the primary between-level mediation model without unequal benefits, and report the unequal-benefits specification as a robustness check. At the same time, this robustness pattern suggests that institutional injustice may represent an important regional pathway to mistrust/reserve in its own right and therefore warrants more direct investigation in future research.

Negative perceptions of scientists at the regional level were positively related to conspiracy beliefs (β = .081, p = .004) and yielded a small positive indirect effect on mistrust/reserve (.040, 95% CI [.008, .084]), with a modest positive total effect (.164, 95% CI [.004, .329]). Democracy satisfaction was negatively associated with regional conspiracy beliefs (β = −.195, p < .001) and showed a significant negative indirect effect on mistrust/reserve (−.085, 95% CI [−.152, −.027]), yielding a negative total effect (−.242, 95% CI [−.389, −.097]). Electoral integrity was excluded from the between-level model due to conceptual overlap with other political-legitimacy indicators and concerns about model stability when entered jointly. Religiosity showed a strong positive direct association with mistrust/reserve (β = .232, p < .001) and a positive total effect (.107, 95% CI [.060, .156]), while its indirect component via conspiracy beliefs was small (.007, 95% CI [−.001, .019]). Out-group trust (z) was strongly associated with lower conspiracy beliefs (β = −.174, p < .001), producing a negative indirect effect (−.028, 95% CI [−.051, −.008]) and a near-zero total effect (−.019, 95% CI [−.082, .044]). Threat-oriented worldview showed a positive total association with mistrust/reserve (.426, 95% CI [.070, .779]), although its indirect component was not statistically reliable (.043, 95% CI [−.015, .120]). Economic indicators (purchasing power; gender employment inequality) showed weak and non-robust associations in the revised between model (Table 5).

## V. Discussion

### А. Within (individual) level structural pathways

#### Central mediation path (H1).

Consistent with H1, knowledge **shows** the strongest hypothesized indirect effect and this mediated pathway accounts for a substantial portion of its total association with mistrust/reserve (Table 3). While several covariates also show statistically significant indirect effects, their magnitudes are markedly smaller than the knowledge-mediated pathway.

Informational antecedents exhibit divergent pathways to mistrust/reserve. Passive engagement is associated with lower mistrust primarily through a direct pathway, whereas self-perceived information about scientific discoveries shows offsetting direct and indirect associations that yield a near-zero total effect. In contrast, social media use is positively related to conspiracy thinking and indirectly heightens mistrust via this pathway, even though its overall total association is small. This pattern aligns with evidence that conspiracy-oriented individuals disengage from conventional information channels [91] in favor of informal networks such as social media [30], where conspiratorial narratives and misinformation can be amplified—particularly under epistemic insecurity and motivated reasoning, and in cultural climates characterized by uncertainty avoidance [92,93].

Perceptual variables suggest that mistrust/reserve may differ by its object. Perceived unequal benefits of science and negative views of scientists exert predominantly direct effects on mistrust/reserve, consistent with Goldenberg’s [94] argument that much “science denial” reflects institutional rather than purely epistemic mistrust. In these cases, mistrust appears to function as an attitudinal response to perceived institutional injustice or heightened vigilance toward authority [95], expressing a normative critique in which conspiracy beliefs play a secondary mediating role. Although these strong direct effects underscore their importance as predictors of mistrust/reserve, our primary focus is on the relative indirect effects operating through conspiratorial reasoning. In this regard, the findings support our main hypothesis: conspiratorial reasoning serves an important mediating pathway in the knowledge → mistrust/reserve relationship, whereas for perceived unequal benefits and negative views of scientists, indirect effects via conspiracy are comparatively weaker and their influence is expressed mainly through direct evaluative routes.

By contrast, the indirect pathway observed for perceived vaccine inefficacy indicates that rejecting biomedical consensus is partly routed through conspiratorial reasoning, consistent with work suggesting that conspiracy theories can provide explanatory coherence under epistemic insecurity and contested evidence [96]. Together, these patterns are consistent **at least two pathways through which resistance to science may be expressed**: one rooted in institutional mistrust that operates mainly through direct evaluative judgments, and another characterized by conspiratorial closure that resolves epistemic uncertainty by constructing rigid alternative frameworks—often through distorted or unfalsifiable explanations.

Among ideological and sociocultural factors, religiosity shapes mistrust/reserve primarily through direct normative judgments, with conspiratorial mediation present but comparatively small (Table 3). Although often correlated, religiosity and conspiracy thinking can function as competing frameworks for interpreting power and epistemic authority, especially in polarized contexts [97]. Mistrust may emerge when science is seen as encroaching on domains—such as morality—where religion claims primacy [59]. In contrast, satisfaction with democracy predicts mistrust/reserve largely through conspiratorial pathways (i.e., an indirect effect via conspiracy beliefs with little direct association), consistent with prior work linking institutional evaluations to conspiracy belief. Right-leaning orientation, by comparison, shows both direct and mediated associations with mistrust/reserve, indicating partial mediation rather than an exclusively conspiratorial route (Table 3).

This pattern aligns with research showing that right-wing ideology is more consistently associated with conspiracy thinking, whereas religiosity more directly predicts science mistrust [19]. Within the ideological-attitudinal domain, threat-oriented worldviews and populist preferences show particularly strong combined influence—each exhibiting both direct and conspiracy-mediated associations—highlighting how perceived threat and anti-expert sentiment can promote conspiratorial reasoning and, through it, science mistrust [98]. Although conspiratorial beliefs are rarely modelled explicitly as mediators, their central position in our model is consistent with evidence that conspiracy epistemologies link political discontent, institutional distrust, and opposition to scientific authority [99].

#### Conditional pathways and contextual moderation.

After discussing the structural pathways, we next consider how the knowledge-conspiracy-mistrust path is conditioned by informational, normative, and perceptual contexts, reflecting both moderated mediation and mediated moderation dynamics.

#### Moderated mediation (H2a).

Informational moderators—engagement and perceived information—condition the indirect effect of knowledge within the mediation pathway. When high, they enhance knowledge’s capacity to constrain conspiratorial interpretations; when low, this effect weakens. Consistent with this “epistemic amplifier” account, the Knowledge × Engagement; Information interaction indicates that the protective association of knowledge on mistrust/reserve becomes stronger as perceived information increases (i.e., knowledge is more effectively translated into lower mistrust/reserve under higher informational engagement). While prior work suggests that perceived competence without accuracy can sometimes exacerbate miscalibration [74,100], the present results indicate that higher engagement/information intensifies the protective pathway of knowledge rather than reversing it.

For cultural and ideological moderators, knowledge × religiosity shapes the mediation pathway in a distinct way. Higher religiosity attenuates the protective indirect effect of scientific knowledge via conspiracy beliefs, consistent with the idea that trust in science is filtered through moral and normative commitments that are not reducible to epistemic updating alone. This pattern suggests that, in more religious contexts, the benefits of knowledge for reducing conspiratorial interpretations translate less strongly into lower mistrust/reserve, potentially because alternative normative frameworks remain salient when evaluating scientific authority. This interpretation aligns with work portraying conspiracism as providing meaning and closure under uncertainty [101]; our results indicate attenuation of knowledge’s protective pathway. Religiosity therefore appears to act as both a normative filter and a competing system of moral interpretation in shaping how scientific knowledge translates into (mis)trust.

Perceived inequality, by contrast, is rooted in concrete experiential intuition. In our results it functions less as a reliable **moderator** of the knowledge→conspiracy→mistrust pathway and more as a **strong, largely direct driver** of mistrust/reserve, with a smaller but significant mediated component via conspiracy beliefs (Table 3). In other words, knowledge may remain epistemically relevant, yet its translation into institutional trust can be outweighed when fairness is questioned: perceived injustice can sustain mistrust through evaluative and normative judgments even when conspiracy beliefs are held constant. This pattern is consistent with accounts emphasizing that lived experiences of unfairness can motivate suspicion and meaning-making around institutional motives, especially under uncertainty and low epistemic resources [50]. Compared to religiosity’s abstract moral grounding, perceptions of inequality derive from tangible social experience, operating less as an alternative moral framework and more as an affective cue that heightens vigilance and distrust when institutional fairness is doubted.

#### Mediated moderation (H2b).

In the knowledge × negative perceptions of scientists interaction, our results suggest that the effect on mistrust/reserve is primarily direct rather than mediated by conspiracy beliefs. This is consistent with the idea that, when scientific actors are perceived as untrustworthy, scepticism can be justified without recourse to conspiratorial explanations, shifting evaluation away from alternative explanatory narratives toward actor-focused credibility judgments [102]. Importantly, negative perceptions of scientists do not necessarily imply rejection of scientific knowledge; rather, they can reconfigure the basis of scepticism such that mistrust is grounded in evaluations of scientific representatives and institutions rather than in epistemic opposition to science itself. This interpretation aligns with evidence on the science confidence gap—particularly among less-educated groups—where scepticism is more strongly tied to institutional distrust than to outright rejection of scientific knowledge [65].

#### Cross-level moderation (H2c).

At the cross-level, regional religiosity functions as a normative climate that conditions how individual scientific knowledge translates into mistrust/reserve. In more religious regions, the overall protective effect of knowledge is weaker, indicating a partial decoupling of epistemic competence from trust in scientific institutions. One plausible interpretation is that, in more religious regions, scepticism is more often justified through normative or identity-based evaluations of scientific authority, whereas in less religious regions it may rely more on explanatory narratives such as conspiratorial accounts. Importantly, this cross-level pattern appears to operate primarily through the non-mediated (direct) component of knowledge’s association with mistrust/reserve rather than through large shifts in the conspiracy-mediated pathway, which differs only slightly across regional religiosity. These findings suggest that the relationship between science and religion reflects neither simple coexistence nor a deficit logic, but an interactional dynamic in which epistemic resources and normative climates jointly shape the translation of knowledge into (mis)trust.

Taken together, ideological and cultural factors suggest two distinct logics shaping mistrust (and its mediated vs direct components). In more religious contexts, the protective role of scientific knowledge appears less tightly coupled to institutional trust, consistent with normative and identity-based commitments that can compete with epistemic authority. By contrast, under institutional conditions such as perceived inequality, protective role of knowledge on mistrust is driven strongly and largely directly—consistent with a critical, justice-oriented evaluation of scientific institutions—while conspiratorial mediation plays a secondary role.

### B. Between level (regional) structural path (H3)

At the between level, analyses corroborate H3. Across predictors, regional effects consistently operate by modifying the strength or relevance of the knowledge–conspiracy–mistrust pathway rather than replacing it, a focal indirect pathway in regional science mistrust. However, the very strong between-level associations among some regional knowledge and conspiracy beliefs indicate that these mechanisms are not always cleanly separable and should be interpreted with caution. This strong coupling is not entirely unexpected, given that regional knowledge and conspiracy climate tap related epistemic dimensions of science-related orientations, even if they remain conceptually distinct. Furtermore, at the regional level, the outcome reflects institutional mistrust rather than individual scepticism, emphasizing shared evaluative climates rather than personal doubt. Specific covariate indirect and total effects along this pathway are discussed below.

Regions characterized by more negative views of scientists exhibit higher overall mistrust/reserve primarily through conspiracy climates, suggesting that scepticism about scientific actors can diffuse into broader institutional mistrust via shared conspiratorial meaning-making. Importantly, in sensitivity analyses that included regional perceived unequal benefits of science, this “institutional injustice” climate strongly predicted mistrust/reserve and substantially attenuated the Knowledge → Conspiracy → Reserve mediation, consistent with overlapping between-level variance among perceived unfairness, conspiracy climates, and mistrust/reserve.

Taken together, these patterns indicate that regional mistrust can be sustained both by conspiratorial epistemologies and by broader evaluative judgments about institutional fairness and legitimacy, echoing evidence that unequal distributions of knowledge, authority, and resources corrode trust at both epistemic and normative levels [103].

Parallel to the individual- and cross-level findings, regional religiosity shows a predominantly direct association with mistrust/reserve, with only a weak and non-robust indirect component via regional conspiracy beliefs. This suggests that regional value climates may ground mistrust primarily through broader faith-based orientations and normative evaluations of scientific authority rather than through conspiratorial sense-making.

Political legitimacy appears to shape institutional mistrust/reserve in part by constraining regional conspiracy climates, aligning with prior research [104,105]. Regions with higher democratic satisfaction show lower levels of conspiracism, exhibiting a significant indirect reduction in mistrust/reserve, yielding an overall negative total effect (Table 5). This relationship should nevertheless be interpreted with caution, as democratic satisfaction is plausibly collinear with broader structural features such as economic development, institutional effectiveness, and corruption control [81]. Overall, the pattern is consistent with democracy functioning as a contextual buffer that structures the epistemic environment in which scientific authority is evaluated, rather than operating solely through a single direct pathway [106].

Threat-oriented worldviews and out-group trust relate to science mistrust/reserve through distinct pathways. In contexts of heightened threat sensitivity, mistrust appears to reflect a broader climate of insecurity: conspiracy beliefs may contribute, but the association is driven primarily by non-mediated contextual influences rather than a robust indirect pathway alone. By contrast, out-group trust is strongly and negatively associated with regional conspiracy beliefs, yielding a significant indirect pathway to lower mistrust/reserve, even though the overall total association is close to zero once direct pathways are accounted for. Taken together, these patterns highlight conspiracy thinking as a selective conduit through which intergroup orientations can translate into science-related mistrust/reserve, while threat-based mistrust is more strongly anchored in broader insecurity climates. This aligns with research showing that conspiracy theories—particularly under climates of insecurity—can foster generalized institutional distrust by portraying decision-making processes as fundamentally unfair and illegitimate, even when the conspiracy claims are not directly tied to the institutions in question [107,108].

Regional economic conditions show relatively weak and uneven associations in the revised between-level model. Purchasing power trends in the expected negative direction (higher purchasing power associated with lower conspiracy and lower mistrust/reserve), but these associations are not robust once other regional factors are included, suggesting that economic security may operate more as a background correlate than a primary driver of regional science mistrust [80,109]. Gender employment inequality likewise shows no reliable direct or indirect association with mistrust/reserve in the current specification. Overall, the between-level results are consistent with economic context functioning as a distal background condition [108] rather than a central mechanism shaping regional mistrust/reserve.

## VI. Conclusion & limitations

Taken together, our findings position scientific knowledge neither as a uniformly protective factor, as assumed by deficit models, nor as evidence of stable coexistence between incommensurable epistemologies, as implied by cognitive polyphasia. Instead, knowledge emerges as a consequential epistemic resource whose association with science mistrust/reserve depends on informational, normative, and institutional contexts. Across levels, higher knowledge is consistently linked to lower conspiracy beliefs, and conspiracy beliefs in turn are reliably associated with higher mistrust/reserve, highlighting conspiratorial reasoning as an important pathway through which epistemic resources translate into institutional (mis)trust. At the same time, knowledge’s protective association is not fixed: it is amplified under higher informational engagement and perceived information and is attenuated under higher religiosity and more religious regional climates, underscoring that epistemic competence can be partially decoupled from institutional trust when normative commitments and identity-relevant evaluations are salient.

In this vein, science mistrust/reserve reflects neither simple ignorance nor entrenched epistemic plurality, but contextually patterned pathways through which knowledge, conspiratorial reasoning, and evaluative orientations toward the scientific establishment are configured. By tracing these pathways at both the individual and subnational levels, our analysis moves beyond broad paradigm debates to specify how science mistrust/reserve is structured by cognitive resources and attitudinal factors and by shared political and cultural climates. In doing so, it bridges the explanatory clarity of deficit-oriented accounts—particularly regarding the role of knowledge in constraining conspiratorial interpretations and science scepticism—with the contextual sensitivity of constructivist approaches that emphasize institutional credibility, value conflict, and moralized evaluations of authority. The resulting picture is a middle-range synthesis: the epistemic authority of science is negotiated through the interplay of cognitive resources, sociocultural meanings, and institutional legitimacy.

These insights, however, should be viewed as provisional given several empirical and methodological constraints:

First, the analyses rely on cross-sectional and secondary data, which limits causal inference and the ability to align measurement perfectly with the theoretical constructs under investigation. Although the proposed pathways are theoretically grounded and temporally plausible, longitudinal and experimental designs are needed to capture dynamic feedback processes between knowledge acquisition, conspiratorial beliefs, and mistrust/reserve.

Second, while regional aggregation enables more granular multilevel analysis than national comparisons, between-region associations describe shared contextual climates rather than individual attitudes and should not be interpreted as direct analogues of individual-level processes, raising the usual cautions regarding ecological fallacy. At the same time, regional aggregation may obscure everyday context meaning negotiation and interpretive practices through which science scepticism is constructed, underscoring the value of mixed-method and qualitative research capable of tracing how knowledge, trust, and conspiratorial beliefs are negotiated in specific contexts.

Third, our results should be interpreted with an awareness that model-based estimates are conditional on specification. In multivariable multilevel models, coefficients reflect the association of each predictor *given the others included*; when predictors overlap conceptually and empirically, small specification changes can reallocate shared variance across pathways. Accordingly, our modelling strategy is best understood as a disciplined simplification guided by theory and robustness checks: the most stable conclusions are those that persist across defensible specifications (notably the central knowledge → conspiracy → mistrust/reserve structure), whereas some secondary coefficients are more contingent and should be interpreted cautiously.

Fourth, several modelling and data-processing features warrant additional caution when interpreting specific coefficients. At the between level, some contextual predictors are highly correlated, and we observed overlap/suppression patterns in sensitivity analyses—for example, adding regional perceived unequal benefits of science substantially attenuated the Conspiracy_between → reserve_between association—suggesting that closely related “institutional injustice” climates can absorb variance otherwise attributed to conspiracy beliefs. More generally, this highlights that certain regional constructs may not be cleanly separable and may capture overlapping facets of institutional legitimacy.

Fifth, missing-data decisions and preprocessing steps can influence estimates in multilevel models that combine item-based scaling and structural modelling. Different conventions for handling “Don’t know” responses and other forms of item nonresponse—such as coding DK as incorrect versus treating DK as missing [110]—may yield different distributions of key constructs and can therefore shift downstream path estimates, especially for smaller or more weakly measured effects. Although we adopted a conservative, transparent approach in the final workflow, we acknowledge that alternative defensible treatments of DK responses and missingness could lead to modest differences in some findings, reinforcing the importance of sensitivity analysis and cautious interpretation of more model-dependent coefficients.

Sixth, the present findings also point to the importance of pathways beyond conspiratorial reasoning. In particular, the comparatively strong direct effects of perceived unequal benefits of science and negative views of scientists suggest that institutional injustice may represent important routes to science mistrust/reserve in their own right and deserve more focused attention in future research.

Finally, although the models incorporate a broad set of cognitive, normative, and sociopolitical factors, other influences—such as media ecologies, elite signalling, and issue-specific scientific controversies—may further condition these pathways and help explain cross-context heterogeneity.

More broadly, our results highlight a general lesson about model-based inference in complex social data for future research. From an abductive perspective, model selection is best approached not as the discovery of a single “true model,” but as an iterative process of abductive triangulation in which theory and evidence mutually inform one another: researchers begin from pre-specified hypotheses, evaluate fit and plausibility, examine robustness across defensible specifications, and remain transparent about which findings are stable versus contingent. In this sense, model selection becomes a disciplined way of learning under uncertainty—clarifying mechanisms where evidence converges, while resisting overinterpretation where results depend heavily on modelling choices. Importantly, this triangulation need not be purely quantitative: mixed-method and qualitative evidence can not only help cross-validate whether the constructs and pathways implied by statistical models align with how science mistrust is articulated and negotiated in everyday settings, but also surface productive tensions and interpretive mismatches that refine theory and generate new insights into the socio-cultural organization of epistemic authority of science.

## Supporting information

S1 Appendix Measurement details, item wording, and sensitivity analyses. Full wording of all items used in the analysis, the MIRT measurement models and reliability estimates, and sensitivity checks for the two-stage factor-score approach.
(DOCX)
