# Supplementary material for: Beyond deficit and coexistence: Modeling the knowledge–conspiracy–mistrust configuration in public understanding of science
Source: PLoS One. 2026 Sep 18;21(9):e0341946. doi: 10.1371/journal.pone.0341946 (PMC13588360; doi:10.1371/journal.pone.0341946)
Supplement: Supplementary file 1 [file pone.0341946.s001.docx]

# Appendices

# Supplementary methodological details

This appendix provides additional methodological details supporting the analyses reported in the manuscript: “Beyond Deficit and Coexistence: Modeling the Knowledge–Conspiracy–Mistrust Configuration in Public Understanding of Science”. It documents the data preprocessing procedures, treatment of missing values and “Don’t know” responses, construction of latent variables using multidimensional item response theory (MIRT), and the derivation of factor scores used in the multilevel structural equation models (MSEM). The appendix also reports diagnostic and robustness analyses, including measurement comparability checks across countries, cluster-size diagnostics for the multilevel structure, multiplicity adjustments, and additional model diagnostics. In addition, the appendix includes a table reporting the original survey questions from the Eurobarometer 95.2 questionnaire used to construct the latent variables analyzed in the study.

## Appendix A. Latent variable treatment; Missing data handling; Treatment of Likert indicators

**1. Latent variable treatment: Two-stage MIRT–MSEM estimation strategy**

Ideally, a fully joint framework—estimating measurement models for all constructs simultaneously within the multilevel SEM (CFA-latent-variable MSEM with ordinal/binary indicators)—would in principle provide the cleanest propagation of measurement uncertainty. However, this approach is not currently straightforward for our setting because:

***Mixed indicator types and complex measurement models.*** The constructs analyzed in this study are derived from several item batteries containing both binary and ordinal indicators estimated using multidimensional item response theory (MIRT) models (2PL and graded-response models). Joint estimation of multiple IRT measurement models together with a multilevel structural equation model—including cross-level interactions and an extensive set of covariates—is beyond the scope of standard single-package implementations and would typically require specialized Bayesian frameworks or custom estimation procedures.

***Computational and identification constraints.*** The dataset includes a large number of respondents, many items across multiple batteries, and a multilevel structure spanning 307 regions. Joint estimation of measurement and structural models under these conditions would be computationally demanding and may introduce identification challenges, particularly in the presence of cross-level moderation and large sets of predictors.

***Transparency and reproducibility.*** For these reasons, the analysis adopts a two-stage workflow in which latent constructs are first estimated using MIRT models and the resulting factor scores are subsequently used as inputs in the multilevel structural equation models (see tables 1-5 for factor loadings , table 6 for fit statistics, and original item wordings table 7). This two-stage MIRT → MSEM approach is widely used in applied multilevel research when joint estimation is not feasible. To maintain transparency, we report measurement-model reliability and score precision and evaluate robustness of the structural results through sensitivity analyses.

*The following tables summarize the multidimensional item response theory (MIRT) models used to estimate the latent constructs included in the multilevel structural equation analysis, including factor loadings, communalities, and global model fit statistics where applicable.*

**Table 1. Unequal Benefits of Science and Technology (graded MIRT; 1 factor)**

This construct was estimated using a graded-response MIRT model based on three items capturing perceptions that scientific and technological progress disproportionately benefits privileged groups.

| Item | F1 | h² |
| --- | --- | --- |
| unequal_benefit | 0.821 | 0.674 |
| only_well_off_countries_benefit | 0.867 | 0.752 |
| env_vs_profit | 0.815 | 0.665 |

M2: Statistic cannot be calculated (too few degrees of freedom)

**Table 2. Science Engagement (graded MIRT; 2 factors)**

Science engagement was modeled using a graded-response MIRT model with two correlated dimensions representing active and passive forms of engagement with science-related activities.

| Item | F1 (Active) | F2 (Passive) | h² |
| --- | --- | --- | --- |
| talk_with_family_friends | 0.0580 | 0.8333 | 0.759 |
| watch_documentaries_read_tech_related_publications | -0.0298 | 0.9414 | 0.852 |
| visit_s_t_museums | 0.4086 | 0.4866 | 0.656 |
| study_s_t_in_free_time_in_courses | 0.5612 | 0.3707 | 0.717 |
| petitions_demonstrations | 0.7059 | 0.1152 | 0.615 |
| attend_public_meetings | 0.8286 | 0.1025 | 0.805 |
| ngo_activities | 0.9284 | -0.0178 | 0.841 |
| contact_public_authorities | 0.9582 | -0.0913 | 0.815 |
| provide_personal_data | 0.6526 | 0.1988 | 0.630 |
| clinical_trials | 0.8074 | -0.0361 | 0.616 |
| lend_computers_processing_power | 0.9189 | -0.1176 | 0.721 |
| active_research | 0.8109 | 0.0777 | 0.744 |

**Table 3. Negative Scientist Perception (2PL MIRT; 1 factor; binary traits)**

Negative perceptions of scientists were modeled using a unidimensional 2PL MIRT model based on binary trait attributions describing scientists.

| Item | F1 | h² |
| --- | --- | --- |
| reliable | 0.864 | 0.746 |
| collaborative | 0.690 | 0.476 |
| narrow_minded | 0.561 | 0.315 |
| bad_at_communicating | 0.434 | 0.188 |
| honest | 0.845 | 0.714 |
| arrogant | 0.594 | 0.353 |
| altruistic | 0.399 | 0.159 |
| immoral | 0.600 | 0.359 |
| intelligent | 0.638 | 0.408 |
| know_best_what_is_good_for_people | 0.503 | 0.253 |

**Table 4. Science Attitudes (Reserve/Promise/Deference; graded MIRT; 3 factors)**

Science attitudes were estimated using a graded-response MIRT model with three dimensions corresponding to Reserve, Promise, and Deference orientations toward science**.**

| Item | F1 (Reserve) | F2 (Promise) | F3 (Deference) | h² |
| --- | --- | --- | --- | --- |
| science_complicated | 0.24659 | 0.06136 | 0.509390 | 0.448 |
| science_not_important_in_daily_life | 0.08290 | 0.00232 | 0.744864 | 0.618 |
| scientists_spend_sufficient_time_to_explain | -0.10442 | 0.41626 | 0.204739 | 0.225 |
| would_like_to_learn_more | 0.15967 | 0.40998 | -0.338892 | 0.230 |
| s.t_can_sort_out_any_problem | -0.00267 | 0.71935 | -0.015273 | 0.514 |
| no_limit_to_what_science_investigates | -0.06285 | 0.63261 | 0.036659 | 0.404 |
| scientists._power | 0.75099 | 0.00679 | 0.044836 | 0.597 |
| new_inventions_against_harmful_s.t_consequences | 0.09307 | 0.64979 | 0.000797 | 0.440 |
| younger_gen_well.informed | 0.02845 | 0.63446 | -0.124736 | 0.396 |
| nat_resources_inexhaustible | 0.05010 | 0.64850 | 0.199771 | 0.514 |
| future_generations | -0.05218 | 0.62901 | -0.180561 | 0.402 |
| artif_intel_creates_more_jobs | -0.12466 | 0.58279 | 0.114286 | 0.364 |
| not_enough_faith | 0.53072 | 0.09780 | 0.200047 | 0.440 |
| human_rights_threat | 0.66555 | -0.10006 | -0.088119 | 0.401 |
| makes_life_change_too_fast | 0.69006 | 0.08940 | -0.036734 | 0.471 |
| no_longer_trust_scientists | 0.72976 | -0.10398 | -0.069866 | 0.493 |
| very_specific_perspectives | 0.67673 | -0.02049 | 0.008797 | 0.462 |
| problems_too_complex | 0.61700 | 0.04863 | 0.184382 | 0.526 |
| scientists_accountable_for_misuse | 0.49712 | 0.09328 | 0.166441 | 0.369 |

**Table 5. Knowledge and Conspiratorial Reasoning (2PL MIRT; confirmatory 3-factor; constrained version)**

| Item | BasicS03 | KnowS03 | ConsS03 | h² |
| --- | --- | --- | --- | --- |
| earlst_human_beings |  | 0.492 | 0.247 | 0.303 |
| moving_continents | 0.917 |  |  | 0.840 |
| antibiotics |  | 0.621 |  | 0.386 |
| oxygen_from_plants | 0.345 |  |  | 0.119 |
| laser |  | 0.650 |  | 0.423 |
| world_population |  | 0.523 |  | 0.273 |
| human_spec_developm | 0.412 |  | 0.193 | 0.207 |
| climate_change_cause |  |  | 0.338 | 0.115 |
| cancer_cure_hidden |  |  | 0.816 | 0.666 |
| viruses_control_freedom |  |  | 0.907 | 0.822 |

**Table 6. Summary of MIRT measurement model fit statistics**

Table A6 summarizes global fit statistics for all multidimensional item response theory (MIRT) measurement models used to derive latent factor scores included in the multilevel structural analysis.

| **Model** | **Model type** | **M2** | **df** | **RMSEA** | **RMSEA 90% CI** | **SRMSR** | **TLI** | **CFI** |
| --- | --- | --- | --- | --- | --- | --- | --- | --- |
| Unequal benefits | Graded MIRT (1 f) | – | – | – | – | – | – | – |
| Science engagement | Graded MIRT (2 f) | 3384.46 | 19 | 0.069 | [0.067, 0.071] | 0.062 | 0.954 | 0.979 |
| Negative scientist perceptions | 2PL MIRT (1 f) | 9564.96 | 35 | 0.087 | [0.085, 0.088] | 0.075 | 0.820 | 0.860 |
| Science attitudes | Graded MIRT (3 f) | 5141.78 | 60 | 0.048 | [0.047, 0.050] | 0.039 | 0.929 | 0.962 |
| Knowledge–conspiracy battery | 2PL MIRT (3 f) | 2920.46 | 31 | 0.051 | [0.049, 0.053] | 0.056 | 0.915 | 0.941 |

**Note.** Fit statistics are not reported for the Unequal Benefits scale because the model is just-identified (three indicators), which yields zero degrees of freedom and prevents calculation of global fit indices.

Factor scores derived from these MIRT models were used as observed variables in the multilevel structural equation models reported in the main text.

**2. Missingness, DK handling, and factor-score uncertainty**

Missingness in observed covariates was addressed using random-forest imputation (*missRanger*), incorporating auxiliary variables capturing demographics, political orientation, science engagement, and survey paradata. The analysis does not assume that missing data are Missing Completely at Random (MCAR). Instead, for observed covariates imputed using *missRanger*, missingness is treated as plausibly Missing at Random (MAR), conditional on the observed predictors included in the imputation models. These predictors comprise demographic characteristics, socioeconomic indicators, measures of political orientation, science engagement variables, and survey paradata used as auxiliary variables in the imputation procedure.

Descriptive diagnostics of missingness patterns were examined, including variable-level missingness rates and associations between missingness and observed covariates. These diagnostics provide supporting evidence for the plausibility of the MAR assumption. At the same time, MAR is ultimately recognized as an untestable assumption, because it concerns relationships involving unobserved data.

***Handling “Don’t know” (DK) responses to knowledge items*** remains a debated issue in the literature. For example, Mondak [92] argues that DK responses are conceptually distinct from incorrect responses and therefore should be coded as missing. However, such approaches remain contested and rely on additional assumptions about the missingness mechanism underlying DK responding. Within an IRT → multilevel SEM analytical pipeline, treating DK responses as missing also introduces further modeling choices, including whether to (i) leave DK responses as missing, thereby reducing usable item information; (ii) impute item responses under MAR assumptions using categorical imputation procedures; or (iii) treat DK responses as incorrect.

By contrast, common practice is to code DK responses on knowledge items as incorrect (0/False) prior to MIRT estimation. This is a conservative and widely used convention for objective knowledge items and avoids generating imputed “factual answers” through model-based filling-in. It also reduces dependence on assumptions about DK missingness and avoids introducing imputed factual responses into the knowledge items. Considering both approaches, DK treatment should therefore be understood as an analytic choice rather than a uniquely correct specification. We considered both approaches and, after conducting sensitivity analyses, decided to follow the second, more conservative approach. Robustness was evaluated by comparing results obtained under this coding strategy with results from the alternative preprocessing specifications described above.

***For all other latent-variable item batteries*** (graded or binary), DK or nonresponse remained coded as missing (typically <5%) and was handled as missing data at the measurement stage.

***No item-level imputation for MIRT indicators.*** Item-level indicators used in the MIRT measurement models were not imputed, and MIRT factor scores were not directly imputed. Instead, factor scores were computed from the observed item responses (including DK = 0 coding for the knowledge battery) and subsequently used as observed inputs in the multilevel structural equation models.

***Factor-score uncertainty.*** In this study, the structural models treat the resulting factor-score components (within-person centered and between-region means) as observed inputs without explicit correction for score error. This scores-as-observed approach follows a transparent two-stage MIRT → MSEM workflow, which is common in applied multilevel research when joint estimation of complex measurement models within multilevel SEM is not feasible. However, because factor scores contain measurement error, treating them as observed inputs may attenuate some associations (bias toward zero), making smaller effects more difficult to detect. Results should therefore be interpreted cautiously, and alternative specifications incorporating measurement-error corrections are reported as sensitivity checks.

**3. Quantitative missingness summary (observed covariates + item batteries)**

1) *Sample sizes and clustering:* Total individual N (raw analytic frame): 35,882; Analytic N used in MSEM (lavaan input): 35,075; Number of clusters (regions) in MSEM analytic file: 308. For the observed-covariate source frame (prior to imputation/merging): N: 37,079; J: 309 regions. Minor differences in sample size and number of clusters across data frames reflect merging of multiple sources, construction of derived variables, and the listwise requirements of the final modeling dataset.

*2) Observed covariates (pre-imputation)*

Across the observed covariates used in the MSEM, overall missingness in the observed-covariate source frame was 1.571%, with variable-level missingness ranging from 0% to 6.098%. Highest-missing observed covariates:

- Right/left ideology (right): 6.10%
- Education (edu_numeric): 4.44%
- Vaccine inefficacy (ineff_vac): 3.91%
- Other observed covariates were ≤ ~1.1% missing (many were ~0%).

All other observed covariates had missingness levels of approximately 1.1% or lower, with many variables having essentially no missing data. After preprocessing and merging into the final MSEM analytic dataset, missingness in the modeled variables was negligible (overall ≈ 0.006% across inspected variables). Residual missingness was concentrated in reserve_cwc (0.092%), while other key modeled variables (e.g., Knowledge_cwc, Conspiracy_cwc, and inspected covariates) were essentially complete.

*3) Objective knowledge items and “Don’t know” prevalence*

“Don’t know” (DK) responses were relatively common in the objective knowledge battery, which were treated as 0/False but not as missing. Before recoding as 0, DK responses account for approximately:

- DK as a share of item responses: 17%
- % of respondents with ≥ 1 DK: 62.24%

In the primary preprocessing strategy used for the measurement models, DK responses in the objective knowledge battery were coded as incorrect (0/False) prior to MIRT estimation. Consequently, the item matrix used for knowledge scoring contains no missing values by construction. The DK prevalence statistics above are reported to document the substantive scope of this recoding decision.

**4. LV Item-battery missingness feeding MIRT scales**

All LV item batteries below were processed with explicit NA handling prior to scoring; missingness is reported at the item-response level and person level,

*Unequal Benefits of Science (3 items; final scoring set):*

- Response-level missingness: 1.60%
- % respondents with ≥1 missing: 2.59%
- % respondents with all 3 missing: 0.89%
- Item-level missingness: env_vs_profit 1.73%, only_well_off_countries_benefit 1.61%, unequal_benefit 1.47%
- Passive Engagement (4 items)
- Response-level missingness: 0.73%
- % respondents with ≥1 missing: 1.50%
- % respondents with all 4 missing: 0.31%
- Item-level missingness: ~0.67–0.81% per item

*Negative Scientist Perceptions (10 items):*

Response-level missingness: 9.84%

- % respondents with ≥1 missing: 26.14%
- % respondents with all 10 missing: 2.20%
- Highest-missing items: altruistic 14.79%, honest 12.41%, immoral 11.49%, arrogant 10.31% (others lower; intelligent lowest at 3.64%)
- Reserve / Science mistrust outcome items (8 items; retained scoring set)
- Response-level missingness: 2.19%
- % respondents with ≥1 missing: 7.89%
- % respondents with all 8 missing: 0.29%

Highest-missing items: very_specific_perspectives 3.04%, problems_too_complex 2.66%, human_rights_threat 2.52% (lowest: makes_life_change_too_fast 1.09%)

*Item indicators in the primary workflow.* Item-level indicators used in the MIRT measurement models were not imputed. In the objective knowledge battery, DK responses were coded as incorrect (0/False) prior to estimation. For all other item batteries, DK or nonresponse responses were retained as missing (typically <5%). Also, MIRT factor scores were not directly imputed.

**5. Sensitivity analyses and robustness checks**

The substantive conclusions of the analysis—particularly the Knowledge → Conspiracy → mistrust/reserve pathway—were not materially altered by differences in DK handling or by clarifications in the two-stage measurement–structural modeling workflow. Coding DK responses as incorrect for objective knowledge items reduces reliance on assumption-dependent DK imputation and provides a transparent basis for subsequent multilevel structural equation modeling.

To assess whether key inferences depend on preprocessing choices, the focal pathways were compared across alternative preprocessing specifications. One specification coded DK responses in the objective knowledge battery as incorrect and used factor scores derived from observed item responses as inputs in the multilevel models. An alternative specification treated DK responses as missing, explored item-level multiple imputation prior to MIRT scoring, and incorporated measurement-error corrections for selected factor scores.

Results from these specifications yielded substantively similar conclusions for the primary Knowledge → Conspiracy → mistrust/reserve pathway. Some secondary coefficients showed greater sensitivity to specification choices and are therefore interpreted with appropriate caution.

**6) Treatment of Likert and other ordinal indicators**

Likert-type and other ordered-response batteries (including items with 4-, 5-, or wider category scales) were modeled as ordinal at the measurement stage using graded-response MIRT models. The multilevel SEM was then estimated on the resulting continuous factor-score inputs (within- and between-components), rather than on the raw ordinal items; accordingly, ML/robust ML estimation applied to continuous score distributions rather than to ordinal indicator modeling within lavaan. Single-item observed Likert-type variables included as covariates were treated as approximately continuous in the structural model, consistent with common practice for ordered-response variables with at least 4–5 categories when used as observed predictors rather than latent indicators. The three binary items were likewise included as observed covariates and treated as approximately continuous for estimation purposes as a pragmatic simplification, although this should be understood as an approximation given their dichotomous response format. Where relevant, monotonicity diagnostics were also examined for ordered-response batteries to confirm ordinal scale structure.

**7) Remaining limitations**

Any missing-data strategy involves assumptions. Coding DK as incorrect is conservative and aligns with standard practice for objective knowledge batteries, but it may understate partial knowledge for some respondents. Conversely, DK-as-missing approaches aim to separate uncertainty/response style from knowledge but require stronger assumptions about DK missingness and additional modeling choices when combined with IRT scaling and multilevel modeling. Likewise, treating factor scores as observed inputs in MSEM can attenuate associations, particularly for constructs with modest reliability; we therefore report reliability/score precision and interpret secondary coefficients cautiously, emphasizing robustness of the central findings across reasonable alternative specifications.

## **Appendix B. Measurement invariance / DIF**

Cross-regional interpretation of latent constructs requires evidence of measurement invariance/comparability. As previously highlighted, the present study uses a two-stage MIRT → MSEM workflow in which multiple item batteries with mixed indicator types are first modeled using multidimensional item response theory (MIRT; 2PL models for binary indicators and graded-response models for ordinal indicators). The resulting factor scores are then decomposed into within- and between-region components and used as inputs in multilevel structural models.

In this setting, standard multi-group CFA invariance testing is not the most natural framework. First, the constructs are estimated using MIRT measurement models rather than linear CFA measurement models. Second, the structural model includes multilevel decomposition, a large set of covariates, and interaction terms, making fully joint item-level estimation within a multilevel SEM computationally demanding in conventional SEM software. Third, invariance testing across a large number of regions (307) would be difficult to implement and poorly powered for many small clusters. For these reasons, measurement comparability was evaluated at the level most directly relevant to the analytical pipeline: the stability of MIRT-derived factor scores across countries.

As an approximate comparability check, pooled-model factor scores were compared with country-specific factor scores estimated separately within each country under the same MIRT specifications. Within-country correlations between pooled and country-specific scores were then calculated. This score-concordance approach evaluates whether the latent ordering implied by the pooled calibration is preserved within countries, which is the key requirement for using pooled scores in downstream multilevel modeling.

Results indicate strong practical comparability, particularly for the outcome scale. For the Reserve (mistrust) battery (graded-response model), pooled–country score concordance was extremely high across countries (r = .899–.999), with nearly all countries ≥ .98 and most ≥ .99, indicating strong stability of the outcome score across national contexts. For the knowledge/conspiratorial reasoning battery (2PL model), concordance was generally high across countries (often ≥ .95), although a small number of contexts showed somewhat lower—yet still moderate to high—concordance (approximately r ≈ .81–.83). This pattern is consistent with the known cross-national sensitivity of certain knowledge and conspiratorial items (e.g., health - and evolution-related misconceptions and explicitly conspiratorial statements), and cross-national equivalence for this battery is therefore interpreted as approximate rather than strict.

Overall, the results indicate that the MIRT-derived scores used in the multilevel structural models are broadly stable across countries—particularly for the key outcome construct—while suggesting cautious interpretation of cross-national contrasts for the knowledge/conspiratorial battery in a small number of lower-concordance contexts.

## **Appendix C. Cluster justification for MSEM**

**Level-2 structure and cluster sizes.**

The analysis was estimated with respondents nested within  **307 regions**  (Level 2; total  **N = 35,882** ). Cluster sizes were heterogeneous (min =  **3** , median = **87** , mean =  **117** , IQR =  **118** , 5th percentile ≈ **17** , 95th percentile ≈ **305** , max = **608** ). The proportion of small clusters was: **6.2%** with n < 20, **15.3%** with n < 30, **29.6%** with n < 50, and **56.0%** with n < 100. These descriptives indicate meaningful between-region structure while also motivating sensitivity checks for sparse clusters.

**Evidence of nontrivial clustering (ICCs).**

Intraclass correlations (from a null multilevel model) indicated substantive regional clustering: ICC = .**269** for Conspiracy and ICC = .**221** for mistrust/reserve, supporting a multilevel specification and within–between decomposition.

Within–between differentiation (“Hausman-spirit” checks).

In a basic within–between mediation model, robust Wald tests confirmed that key between-level parameters were nonzero and that within- and between-level effects differed significantly. Specifically, the between-level Knowledge → Conspiracy path was nonzero ( **χ²(1) = 1092.97, p < .001** ), and the between-level direct Knowledge → mistrust/reserve path was also nonzero ( χ²(1) = 6.01, p = .014 ). In the spirit of a Hausman-type check, equality constraints across levels were rejected (testing a**_w = a_b**  and **c_w = c_b** : **χ²(2) = 206.80, p < .001** ), indicating that within- and between-level associations are not interchangeable and supporting the MSEM approach.

**Sensitivity to small cluster sizes.**

To assess whether results were driven by sparse regions, we refit a reduced version of the multilevel model (core Knowledge→Conspiracy→mistrust/reserve pathways, plus within-level interaction terms) after excluding regions with **n < 30** (47 of 307 regions). Parameter estimates were essentially unchanged. For example, the within-level Knowledge → Conspiracy path remained identical ( **a_w = −0.524**  in both samples), as did the within b-path ( **b_w = 0.412** vs 0.413 ) and the within total effect ( **tot_w = −0.432** vs **−0.434** ). Between-level mediation was likewise stable ( **a_b = −0.712** vs −0.716 ; **b_b = 0.372** vs **0.368** ; **ind_b = −0.265** vs **−0.263** ; **tot_b = −0.579** vs **−0.571** ). Interaction coefficients also showed negligible differences across specifications. Within-level moderation terms were similarly stable (e.g., c_kno_eng = −0.053 vs −0.051; c_cons_eng = 0.078 vs 0.080; c_kn_cross = 0.080 vs 0.079; c_kno_neg = 0.054 vs 0.053). Together, these checks indicate that the substantive conclusions are not driven by a small number of very small clusters and that decomposing effects across levels is appropriate for these data.

## **Appendix D. Multiplicity adjustment (Benjamini–Hochberg / FDR)**

To address the concern about multiple testing, we applied the Benjamini–Hochberg false discovery rate (BH/FDR) procedure to adjusted p-values **within three pre-specified families of tests** derived from the multilevel SEM output: (i) within-level regressions (all “~” paths at Level 1), (ii) between-level regressions (all “~” paths at Level 2), and (iii) defined parameters (all “:=” effects, including indirect effects, total effects, and moderated-mediation indices). BH/FDR adjustment was applied within each family (Level 1 regressions: *n* = **90**; Level 2 regressions: *n* = **22**; defined parameters: *n* = **93**). At α = .05, **74/90** Level-1 paths, **10/22** Level-2 paths, and **75/93** defined parameters remained significant after BH adjustment.

**Summary of results under BH/FDR.**
**Within level (Level 1).** Substantive conclusions were unchanged after BH/FDR adjustment. The focal mediation pathway (Knowledge → Conspiracy; Conspiracy → mistrust/reserve; and the indirect effect of Knowledge through Conspiracy) remained statistically robust. Most substantive predictors and their indirect/total effects remained significant after adjustment. A limited set of secondary effects did not survive BH/FDR, including the total effect of social media (while its indirect effect remained significant), the mediated-moderation indirect effect for the Knowledge×negative-scientist interaction, and the unequal-benefits moderated-mediation index.

**Between level (Level 2).** The core between-level mediation relationships remained robust after BH/FDR adjustment, including the Knowledge_between → Conspiracy_between path, the Conspiracy_between → mistrust/reserve_between path, and the direct Knowledge_between → mistrust/reserve_between path. Several secondary contextual paths were not robust after BH/FDR adjustment and are treated as exploratory.

**Defined parameters (indirects/indices).** The within-level indirect and total effects for Knowledge remained significant after BH/FDR. At the between level, the indirect effect of Knowledge_between through Conspiracy_between remained significant after BH/FDR. Among moderated-mediation indices, indices for engagement, information, religiosity, and cross-level moderation remained significant after BH/FDR, whereas the unequal-benefits moderated-mediation index did not.

Overall, BH/FDR adjustment supports the robustness of the primary conclusions while indicating that a small subset of secondary effects should be interpreted with caution.

## **Appendix E. Model diagnostics and model comparison.**

We report global fit indices and information criteria for three models representing the analytic workflow: (i) a null model used to estimate ICCs, (ii) a basic within–between mediation model (Knowledge → Conspiracy → mistrust/reserve), and (iii) the full multilevel SEM. Because these models are not all fit to the same set of observed variables and were estimated with ML, χ² difference testing across them is not appropriate; we therefore compare models using AIC/BIC/SABIC and standard fit indices. The null model (df=2) fits poorly as expected (χ²=4595.39; RMSEA=.253), consistent with substantial clustering. The basic mediation model is just-identified (df=0), so its perfect fit indices (CFI=1.00; RMSEA=0) are a property of zero degrees of freedom rather than evidence of superior model fit. Our primary diagnostics therefore focus on the full model, which shows good overall fit (df=76; χ²=2202.28; CFI=.955; TLI=.875; RMSEA=.029; SRMRwithin=.013; SRMRbetween=.008), alongside information criteria (AIC=2,238,061; BIC=2,242,415; SABIC=2,240,775).

## **Appendix F. Original Survey Items Used to Construct Latent Variables**

**Table 7. Original Survey Items Used to Construct Latent Variables**

| **Construct** | **EB Code** | **Question Wording** |
| --- | --- | --- |
| **Science Knowledge / Conspiratorial Reasoning** | QA20_1 | The earliest humans lived at the same time as the dinosaurs |
|  | QA20_2 | The continents on which we live have been moving for millions of years and will continue to move in the future |
|  | QA20_3 | Antibiotics kill viruses as well as bacteria |
|  | QA20_4 | The oxygen we breathe comes from plants |
|  | QA20_5 | Lasers work by focusing sound waves |
|  | QA20_6 | The world's human population is currently more than 10 billion |
|  | QA20_7 | The methods used by the natural sciences and the social sciences are equally scientific |
|  | QA20_8 | Human beings, as we know them today, developed from earlier species of animals |
|  | QA20_9 | Climate change is for the most part caused by natural cycles rather than human activities |
|  | QA20_10 | The cure for cancer exists but is hidden from the public by commercial interests |
|  | QA20_11 | Viruses have been produced in government laboratories to control our freedom |
| **Science Attitudes** | QA9_1 | Science is so complicated that I do not understand much about it |
|  | QA9_2 | In my daily life, it is not important to know about science |
|  | QA9_3 | Scientists spend sufficient time meeting people like me to explain their work |
|  | QA9_4 | I would like to learn more about scientific developments in places like town halls, museums and libraries |
|  | QA9_5 | The results of publicly funded research, such as scientific articles and data, should be made available online free of charge |
|  | QA9_6 | Young people's interest in science is essential for our future prosperity |
|  | QA9_7 | Science and technology can sort out any problem |
|  | QA9_8 | There should be no limit to what science is allowed to investigate |
|  | QA9_9 | New inventions will always be found to counteract any harmful consequences of scientific and technological development |
| **Science Engagement Engage Passive** | QA14_1 | Talk about science and technology-related issues with family or friends |
|  | QA14_2 | Watch documentaries, or read science and technology-related publications, magazines or books |
|  | QA14_3 | Visit science and technology museums |
|  | QA14_4 | Study science and technology-related issues in your free time, for instance on a face-to-face or online course |
| **Engage active** | QA14_1 | Talk about science and technology-related issues with  family or friends |
|  | QA14_2 | Watch documentaries, or read science and technology-related publications, magazines or books |
|  | QA14_3 | Visit science and technology museums |
|  | QA14_4 | Study science and technology-related issues in your free time, for instance on a face-to-face or online course |
|  | QA14_5 | Sign petitions or join demonstrations on science and technology matters such as nuclear power, biotechnology, the environment or climate change |
|  | QA14_6 | Attend public meetings or debates about science and technology |
|  | QA14_7 | Take part in the activities of a non-governmental organisation dealing with science and technology related issues |
|  | QA14_8 | Contact public authorities or political leaders about science and technology-related issues |
|  | QA14_9 | Provide personal data for scientific research |
|  | QA14_10 | Take part in clinical trials |
|  | QA14_11 | Lend your computer's processing power to contribute to research on complex scientific questions |
|  | QA14_12 | Actively take part in scientific projects by developing research questions, collecting data, discussing the findings with others, etc. |
| **Scientist Perceptions** | QA12a_1 | Reliable |
|  | QA12a_2 | Collaborative |
|  | QA12a_3 | Narrow minded |
|  | QA12a_4 | Bad at communicating |
|  | QA12a_5 | Honest |
|  | QA12a_6 | Arrogant |
|  | QA12a_7 | Altruistic |
|  | QA12a_8 | Immoral |
|  | QA12a_9 | Intelligent |
|  | QA12a_10 | Know best what is good for people |
| **Perceived Unequal Benefits of Science** | QA17_1 | Science and technology do not really benefit people like you |
|  | QA17_2 | Science and technology could be used to improve everyone's lives, but in practice they mostly improve the lives of people who are already better off |
|  | QA17_3 | Science and technology could be used to improve living conditions in less developed countries, but in practice they mostly improve living conditions in well-off countries |
| ***Observed Variables*** |  |  |
| **Religiosity** | D90.1 | On a scale of 1 to 10, how religious or spiritual do you consider yourself? |
| **Left–right political orientation** | D1 | In political matters people talk of "the left" and "the right". How would you place your views on this scale? |
| **Populist decision-making** | QA7_4 | Public opinion should be the main concern when making decisions about science and technology |
| **Use of social media as a main source of science information** | QA4a_7 | Online social networks and blogs (e.g. video hosting websites) |
| **Self-perceived information about scientific discoveries (information)** | QA3_2 | New scientific discoveries and technological developments |
| **Perceived inefficacy of science** | QA8a_5 | Vaccines and combatting infectious diseases |
|  | QA11_4 | Scientists should not intervene in political debate when decisions ignore scientific evidence |
|  | QA11_5 | Scientists should intervene in political debate to ensure that decisions take into account scientific evidence |
